# Combine mitochondrial-targeted gene therapy and chemotherapy to treat triple-negative breast cancer

**DOI:** 10.1186/s13046-025-03572-8

**Published:** 2025-12-29

**Authors:** Tanvi Varadkar, Zhuoxin Zora Zhou, Jiashuai Zhang, Anusua Sarkar, Zhantao Du, Srijita Chowdhury, Hwayeon Lim, Lufang Zhou, Xiaoguang Margaret Liu

**Affiliations:** 1 Department of Chemical and Biomolecular Engineering, The Ohio State University, Columbus, OH USA; 2 Department of Biomedical Engineering, The Ohio State University, Columbus, OH USA; 3 Comprehensive Cancer Center, The Ohio State University, Columbus, OH USA; 4 151 West Woodruff Avenue, Columbus, OH 43210 USA

**Keywords:** Mitochondrial targeting, MAb-Exo-AAV, PARPi, Combined therapy, TNBC treatment

## Abstract

Triple-negative breast cancer (TNBC) is an aggressive and chemotherapy resistant subtype with high metastatic potential and frequent recurrence after standard treatment. While poly (ADP-ribose) polymerase inhibitors (PARPi) show efficacy in TNBCs with DNA repair deficiencies, only ~ 20% of patients respond, underscoring the need for more effective therapies. Mitochondria, as central regulators of cancer cell survival, present a compelling therapeutic target. Here, we introduce a novel gene-chemotherapy combining our mitochondria-targeted luminoptogenetics technology (cmLumiOpto), which directly disrupts mitochondrial membrane potential and induces cancer cell death, with PARPi to enhance TNBC treatment outcomes. To achieve targeted delivery, we conjugated a high-affinity anti-CD276 monoclonal antibody (mAb) that selectively binds human and mouse TNBCs to an exosome-associated adeno-associated virus (mAb-Exo-AAV). In vitro studies confirmed successful transfection, internalization, and functional expression of cmLumiOpto, leading to significantly enhanced cytotoxicity when combined with PARPi. In vivo, the combination therapy achieved a 95–100% reduction in tumor burden, suppressed patient-derived xenograft growth, and inhibited metastasis in four TNBC mouse models. Post-treatment analyses confirmed mitochondrial depolarization, downregulation of DNA replication, cytokine upregulation, and immune cell infiltration in tumor. These findings highlight the potential of mitochondria-targeted gene therapy combined with chemotherapy as a powerful and innovative strategy for TNBC treatment.

**Supplementary Information:**

The online version contains supplementary material available at 10.1186/s13046-025-03572-8.

## Statement of significance

Synergism of mitochondria-targeting cmLumiOpto gene therapy with systemic chemotherapy PARPi offer an effective therapy for TNBCs.

## Introduction

Triple-negative breast cancers (TNBCs; HER2^-^, ER^-^, PR^-^) are highly aggressive, metastatic, and heterogeneous, accounting for 15–20% of all breast cancer cases. Despite advances in oncology, TNBCs remain among the most challenging malignancies to treat, with recurrence rates exceeding 50% and poor survival outcomes following primary therapy. Standard chemotherapeutic regimens, such as anthracycline-taxane-based treatments, remain the cornerstone of TNBC management [1–4]. However, these therapies frequently fail to achieve long-term disease control, leading to tumor relapses and therapeutic resistance. Single-agent therapies, such as chemotherapy and monoclonal antibodies (mAbs), have shown limited efficacy in recurrent and metastatic TNBC [5–8]. In contrast, combinatorial strategies have shown greater promise. Notably, the integration of immunotherapy with chemotherapy, exemplified by the combination of Atezolizumab (a PD-L1 immune checkpoint inhibitor) and Abraxane (nab-paclitaxel), has introduced a novel therapeutic avenue for PD-L1^+^ TNBC [9–11], highlighting the potential of immune-based approaches. In addition, the FDA-approved antibody-drug conjugate sacituzumab govitecan, an anti-Trop-2 mAb linked to the topoisomerase I inhibitor SN-38, has emerged as an effective option for patients with refractory TNBCs [12–14]. Despite these advances, current conventional and targeted therapies remain insufficient for highly aggressive TNBCs. The primary challenges include early metastatic spread [15], tumor heterogeneity [16–18], low response rates, and the emergence of drug resistance over prolonged treatment durations [19, 20]. Given these obstacles, there is a critical need for innovative therapeutic strategies that can improve patient outcomes.

Mitochondria play a central role in cellular metabolism, proliferation, and apoptosis, making them a promising therapeutic target in cancer [21]. A key vulnerability lies in the inner mitochondrial membrane (IMM) potential (ΔΨ_m_), whose sustained and irreversible collapse drives cells toward apoptosis [22]. To exploit this vulnerability, we recently developed cmLumiOpto, an advanced cancer mitochondrial-targeted luminoptogenetics system that utilizes endogenous Nanoluciferase bioluminescence to activate light-gated cationic rhodopsin channels (CoChR) in the IMM [23]. This system enables controlled, dose-dependent mitochondrial depolarization *via* ViviRen luciferin, with prolonged ΔΨ_m_ collapse leading to persistent DNA damage and apoptotic cell death. Notably, cmLumiOpto demonstrated remarkable efficacy in reducing tumor burden and inducing tumor cell death in glioblastoma and TNBC xenograft mouse models.

In a previous study, we constructed an anti-epidermal growth factor receptor (EGFR) mAb tagged exosome-associated adeno-associated virus (mAb-Exo-AAV) using a biosimilar of cetuximab to deliver therapeutic genes [23]. However, The Human Protein Atlas dataset shows that normal human tissues, such as esophagus, placenta, pancreas, kidney, liver, intestine, and reproductive organs, exhibit high EGFR expression. Therefore, off-target risks and safety concerns associated with EGFR mAb-directed cmLumiOpto gene delivery remain a potential issue. CD276 (also known as B7-H3, Uniprot: Q5ZPR3) [24], an immune checkpoint molecule that suppresses natural killer (NK) and T cells responses [25–27], is overexpressed in >80% of breast cancers [28–30], making it an attractive target for TNBC-specific therapy. We recently developed and engineered a novel mAb capable of binding the extracellular domain of transmembrane CD276, enabling precise TNBC targeting. This mAb exhibits high affinity, cancer specificity, plasma stability, and cross-species reactivity, making it a highly effective therapeutic vector [31]. To further enhance tumor specificity, we developed CD276 mAb-Exo-AAV to target and deliver the cmLumiOpto gene under the control of a tumor-selective *cfos* promoter. We hypothesize that this new drug delivery system not only ensures TNBC-specific targeting but also enhances anti-tumor immunity, creating a dual-action therapeutic strategy that is expected to serve as a better gene delivery vehicle compared to EGFR mAb-Exo-AAV.

Our previous studies [23, 32] show that mitochondrial depolarization in cancer cells and other cell types induces DNA damage, suggesting that blocking DNA damage repair could completely eliminate cmLumiOpto-treated TNBC cells. Given the high prevalence of BRCA1/2 inactivation in TNBCs, the FDA has approved poly (ADP-ribose) polymerase inhibitors (PARPi), such as Olaparib, which disrupt DNA repair mechanisms in tumor cells [33–35] for TNBC treatment [36, 37]. However, despite promising clinical trial results in BRCA1/2 wild-type TNBC (NCT02158507) [35], Olaparib alone or in combination with EGFR inhibitor achieves a moderate 24% response rate. Inspired by the DNA damage-inducing capability of cmLumiOpto, we hypothesize that combining the cancer mitochondria-targeted cmLumiOpto, delivered via CD276 mAb-Exo-AAV, with PARPi will lead to a synergistic effect. Specifically, this approach will amplify DNA damage accumulation and apoptotic cell death through the combined action of cmLumiOpto and PARPi, while activating tumoral immunity via CD276 mAb. Together, these mechanisms enable a multi-pronged attack to eliminate the tumor and ultimately improve treatment outcomes for TNBC.

This study aimed to develop and evaluate this combinatorial therapeutic strategy for effectively targeting aggressive TNBCs. The specificity, gene packaging efficiency, and cytotoxic potential of CD276 mAb and mAb-Exo-AAV carrying cmLumiOpto were systematically characterized in vitro. To assess therapeutic efficacy, we established four TNBC mouse models to investigate the impact of cmLumiOpto/PARPi on tumor burden reduction and metastasis inhibition. Additionally, mechanistic insights were explored using Seahorse metabolic analysis, multiplex Luminex assays, RNA-Seq, and other analytical approaches. The findings provide compelling evidence supporting the clinical translation of this targeted therapy and underscore its potential to improve treatment outcomes in patients with highly aggressive TNBC.

## Materials and methods

### Cell lines and culture media

Viral Production Cell 2.0 (VPC) (Gibco, Cat# A49784, RRID: RRID: CVCL_0045, Grand Island, NY) was cultivated using viral production medium (VPM) supplemented with 4 mM GlutaMAX in shaker flask suspension culture at an agitation speed of 130 rpm. The human TNBC cell lines MDA-MB-231 (ATCC, Cat# HTB-26, RRID: CVCL_0062, Manassas, VA, USA), MDA-MB-468 (ATCC, Cat# HTB-132, RRID: CVCL_0419), and MDA-MB-231-FLuc (GenTarget, Cat# SC059-Puro, RRID: CVCL_YZ80, San Diego, CA, USA) were maintained in DMEM with 10% fetal bovine serum (FBS, v/v) and 1% Pen/Strep in T-flasks. The mouse TNBC 4T1 (ATCC, Cat# CRL-2539, RRID: CVCL_0125) and 4T1-FLuc (ATCC, Cat# CRL-2539-LUC2, RRID: CVCL_5I85) were cultivated in RPMI-1640 with 10% FBS and 1% P/S. The seed culture for CD276 mAb production was kept in SFM medium with 4 mM L-glutamine and 6 g/L glucose [31]. All cell cultures were maintained at 37 °C and 5% or 8% CO_2_ in a humidified incubator (Eppendorf, Enfield, CT, USA). All culture media and supplements were purchased from Fisher Scientific (Waltham, MA, USA) or Gibco, unless otherwise specified. TNBC PDX lines were harvested from donor mice (Jackson Lab, Cat# J000103917, Bar Harbor, ME, USA) or the recipient mice carrying the passaged PDX, freshly frozen and stored in a liquid nitrogen. All commercial lines were authenticated *via* genetics profiling using polymorphic short tandem repeat analysis and tested in house for mycoplasma contamination using PCR amplification of 16 S rRNA gene sequences.

### mAb-Exo-AAV production

Firstly, Exo-AAV was produced in a 2-L stirred-tank bioreactor (Distek, North Brunswick, NJ, USA) using Viral Production Medium (VPM) supplemented with 6 g/L glucose and 4 mM GlutaMAX at 37 °C, pH 7.0, agitation 210 rpm, and DO 40% following previously established protocols [23, 38–40]. VPC cells (viable cell density of 3 × 10^6^ cells/mL, viability of >95%) were co-transfected with three plasmids [23], *AAV-D/J8-cfos-NLuc-2 A-ABCB-CoChR*, *AAV-DJ/8 Rep-Cap*, and *AAV-D/J8 Helper*, at a 1:1:3 with a DNA-to-cell ratio of 0.5 µg per 10^6^ cells. Transfection was mediated using a viral-plex buffer (10% v/v), AAV-MAX transfection reagent (0.6%), booster (0.3%), and enhancer (1%) (Gibco). Exo-AAV was harvested from the spent medium when culture viability declined to 60–80%, centrifuged at 3,000 × g for 20 min at 4 °C, and clarified using a dual-layer regenerated cellulose depth filter (PDK5: 1.5–20 μm, PDE2: 0.2–3.5 μm) (Cytiva, Marlborough, MA, USA). Exo-AAV was purified using liquid chromatography equipped with a 5-mL Cytiva Hiscreen Capto Core 400 column, followed with ultrafiltration using MilliporeSigma Amicon 100 kDa MWCO regenerated cellulose filters, as previously described [23, 41, 42]. Secondly, CD276 mAb was produced in SFM medium using a 2-L stirred-tank bioreactor at 37 °C, Agt 140 rpm, DO 40%, and pH 7.0. The CD276 mAb was purified using liquid chromatography (Bio-Rad, Hercules, CA, USA) with a Bio-Scale Mini UNOsphere SUPrA affinity column. The separated mAb was eluted using a two-phase buffer system, with Phase A (0.02 M Na₃PO₄, 0.02 M Na₃C₆H₅O₇, pH 7.5) and Phase B (0.1 M NaCl, 0.02 M Na₃C₆H₅O₇, pH 3.0) [31, 43–49]. Finally, Exo-AAV was modified using mPEG-DSPE and conjugated with CD276 mAb *via* a DSPE-PEG-NHS linker at a molar ratio of 1:2,680:13,000 (Exo-AAV: mAb: linker). The resulting mAb-Exo-AAV was purified and concentrated using Amicon 100 or 10 kDa MWCO regenerated cellulose filters and stored at −80 °C in 125-mM trehalose formulation buffer.

### Immunohistochemistry (IHC) staining

A TNBC patient tissue microarray (TMA) (Cat# BR1102, US Biomax, Derwood, MD, USA) was stained with an anti-CD276 antibody (Abcam, Rabbit monoclonal, Cat# ab226256, RRID: AB_3069232, 1/500 dilution) following a standard IHC protocol [31]. The stained TMA slide was scanned with Lionheart FX automated microscope (BioTek, Winooski, VT, USA), and images were processed offline with Image J. CD276 expression in each patient tissue sample was calculated using the formula: red_intensity_/blue_intensity_ of TNBC core/red_intensity_/blue_intensity_ of positive core − 1) x100. Receptor expression levels were categorized as follows: high (>0.5, indicating *≥* 50% higher expression than the positive control), medium (−0.3-0.5), and low or no (<−0.3, indicating 30% lower than positive control).

### Nanoparticle tracking analysis

mAb-Exo-AAV samples were buffer exchanged and diluted using PBS with dilution factors of 1:100. The particles were titrated and analyzed using NanoSight Pro (Malvern Panalytical, Malvern, UK). Samples were injected into microfluidics device at a pump perfusion rate of 3 µL/min and imaged with parameters setup of 60 s per capture, 5 captures per sample, 6 camera, detection threshold 5, and Temp 25 °C. Each sample was titrated three times to analyze the size distribution and particle concentration.

### Transmission electron microscopy (TEM)

TEM imaging was performed to assess the morphology and size of mAb-Exo-AAV nanoparticle and free AAV following our previous procedure with modifications [23, 41, 50, 51]. Briefly, purified mAb-Exo-AAV samples were diluted at factors of 1:10, 1:100, and 1:1,000 in PBS buffer, and 10 µL of each sample was deposited onto carbon-coated Formvar grids. Prior to sample loading, grids were glow-discharged for 1 min using a K100X Glow Discharger (Electronic Microscope Sciences, Hatfield, PA, USA). Samples were then negatively stained with 2% uranyl acetate solution for 1 min, followed with two PBS washes and air drying. TEM images were acquired using a Tecnai T12 transmission electron microscope equipped with a CCD camera (Field Electron and Ion Company, Hillsboro, OR, USA) and processed with DigitalMicrograph software (Gatan, Pleasanton, CA, USA).

### qRT-PCR Titration

The Exo-AAV samples were digested with DNase I to extract the packed ssDNA carrying cmLumiOpto gene. RT-PCR analysis was performed to titrate AAV, i.e. nanoluciferase (*NLuc*) genome copy, using the following primers: 5’-ATTGTCCTGAGCGGTGAAA-3’ (forward) and 5’-CACAGGGTACACCACCTTAAA-3’ (reverse). The AAV packing rate in exosome was calculated using genome copy of *NLuc* gene per nanoparticle of Exo-AAV.

### Western blotting

As detailed in our previous publications [23, 52], the lysates of TNBC cells or Exo-AAV samples were loaded to NuPAGE 4–12% gradient Bis-Tris gel for SDS-PAGE electrophoresis (Fisher). Proteins separated on gel were transferred to a methanol activated PVDF membrane with Bio-Rad power supply (Bio-Rad) and blocked using TBS buffer containing 0.1% Tween-20 and 5% fat-free milk. The primary antibodies of CD276 (Cat# ab134161, Abcam), γ-H2AX (Cat# ab2893, Abcam), cleaved PARP (Cat# 9148, Cell Signaling Technology, Danvers, MA, USA), cleaved caspase 3 (Cat# 9661, Cell Signaling), LC3B (Cat# 8899, Cell Signaling), β-actin (Cat# sc-47778, Santa Cruz, CA, USA), and exosome panel of CD9, CD63, HSP70 and calnexin (Cat# ab275018, Abcam) with dilution factor of 1:1,000 or 1:2,000 were applied. The horseradish peroxidase (HRP)-conjugated secondary antibodies (Cell Signaling) and HRP substrate were used to detect the interested protein bands with Odyssey Fc imaging system (LI-COR Biosciences, NE, USA).

### Flow cytometry

To assess the surface binding rate to TNBC cells, CD276 mAb and mAb-Exo-AAV were labeled with fluorescent dyes: Alexa Fluor™ 647 (Life Technologies, part of Fisher) and Sulfo-Cyanine 5.5 (Lumiprobe Life Science Solutions, Hunt Valley, MD, USA), respectively. About 1 × 10^6^ TNBC cells (MDA-MB-231, MDA-MB-468, 4T1) were incubated with 1 µg of CD276 mAb-AF647 or 10 × 10^6^ of mAb-Exo-AAV-Cy5.5 particles at room temperature for 30 min. Surface binding was analyzed using a BD LSR Fortessa flow cytometer (BD Biosciences, San Jose, CA, USA), with a gating strategy set to exclude > 0.5% fluorescent populations of unstained cells. Data were processed and analyzed using FlowJo V5.0 software to determine the surface binding rate.

### Live-cell confocal imaging

TNBC MDA-MB-468 cells expressing GFP were seeded at a density of 1 × 10^5^ cells/mL in a 15-mm glass-bottom dish (Cellvis, Mountain View, CA, USA) and cultured for 6 h. About 10 × 10^5^ particle (ptc)/mL of CD276 mAb-Exo-AAV, labelled with Cy5.5 fluorescent dye, was added to transfect the TNBC cells, followed by incubation at 37 °C and 5% CO_2_ for 24 h. Live-cell images were acquired using a Nikon A1R-HD25 confocal microscope (Nikon, Melville, NY, USA) with 640 nm and 488 nm lasers for Cy5.5 and GFP fluorescence, respectively. Confocal images were analyzed with ImageJ to assess the internalization of mAb-Exo-AAV. To evaluate mitochondrial depolarization, cells treated with cmLumiOpto were stained with MitoView 633 [53] (25 nmol/L) for 15 min and imaged at 635 nm using a Stellaris 5 Confocal microscope (Leica Camera, Teaneck, NJ).

### Seahorse assay

MDA-MB-231 cells treated with mAb-Exo-AAV at multiplicity of infection (MOI) of 100,000 were seeded in a 96- well microplate at a density of 2,000 cells per well. Upon reaching 90% confluence, the following treatments were applied: saline or ViviRen/PARPi. Mitochondrial activity in TNBC cells was assessed using the Seahorse XF Cell Mito Stress Test Kit and quantitated using a Seahorse XF Analyzer (Agilent, Santa Clara, CA, USA) following the manufacturer’s instructions.

### Luminex assay

Chemokines and cytokines in the tumor microenvironment (TME) post treatment were titrated using a Procine Multiplex Luminex assay (Luminex Corporate, Austin, TX, USA). The pre-configured and customized 13-plex assay kit was purchased from R&D Systems (Minneapolis, MN, USA). Tumor tissues (*n* = 4) were dissociated to extract the secreted chemocytokines to perform the assay following manufacturer’s procedure. Fluorescence intensity (MFI) was detected and quantitated using the Luminex MAGPIX (Luminex Corporate) and the raw data were analyzed using XPONENT software.

### In vitro anti-TNBC cytotoxicity assay

Approximately 1 × 10^5^ TNBC (MDA-MB-468 and MDA-MB-231) cells were seeded in 200 µL of medium in 96-well plates. The cultures were treated with saline, CD276 mAb-Exo-AAV carrying cmLumiOpto (MOI: 100,000) and ViviRen (30 µM), PARPi (Olaparib, 20 µM), or cmLumiOpto/PARPi combination, and incubated at 37 °C with 5% CO_2_ for three days. Cell growth and relative viability were assessed using the TACS MTT Cell Proliferation Assay [54, 55].

### In vivo imaging system (IVIS)

Live-animal and ex vivo IVIS imaging was performed to evaluate TNBC targeting specificity of CD276-mAb. Briefly, when tumor volume reached 50–100 mm^3^, 50 µg of mAb labelled with Cy5.5 was intravenously (i.v.) injected through tail vein. After 24 h, luciferin was i.p. injected, and mice were imaged using IVIS Lumina Series III (PerkinElmer, Waltham, MA) with exposure time of 10 s. Both luminescence (FLuc) and fluorescence (Cy5.5) signals were captured. Additionally, major organs and tumors were harvested for ex vivo imaging to validate the biodistribution of CD276 mAb. Beyond distribution analysis, IVIS imaging was also used to monitor in vivo metastasis of TNBC cells expressing FLuc.

### TNBC cell line-derived xenograft model and in vivo treatment

A total of 5 × 10^6^ TNBC MDA-MB-231 cells were injected into the mammary fat pad of 7-week-old NSG (NOD.Cg-Prkdc < scid > Il2rg < tm1Wjl>/SzJ) female mice. When tumor volume reached ~ 75–100 mm^3^, the mice were randomized into six groups (*n* = 6/group). Group 1 received i.v. saline injection as a control. Groups 2–4 were i.v. administrated with CD276 mAb-Exo-AAV weekly at doses of 2 × 10^10^ ptc/kg-BW (low), 10 × 10^10^ ptc/kg-BW (medium) or 30 × 10^10^ ptc/kg-BW (high), followed by i.v. injection of ViviRen (2 mg/kg-BW) daily for three consecutive days. Group 5 received oral administration of 50 mg/kg of Olaparib (PARPi) *via* water bottle feeding. Group 6 was treated with a combination of cmLumiOpto (low dose, 2 × 10^10^ ptc/kg-BW) and Olaparib (50 mg/kg) following the same treatment regime as Groups 2–5. Tumor volumes were measured using a vernier caliper and mouse body weight was monitored two or three times a week. Mice were sacrificed when tumor volume exceeded 1,000 mm^3^, body weight drop by > 20%, or other early removal criteria, such as self-mutilation, inactivity, lethargy, poor response to stimuli, ataxia or ulcerative tumor, were met. In the end of animal study, tumor tissues and vital organs, including brain, heart, lungs, liver, spleen and kidneys, were harvested for H&E staining, IHC staining and biochemical analysis.

### Metastatic model and in vivo treatment

Approximately 2 × 10^6^ mouse TNBC 4T1-FLuc cells and human TNBC MDA-MB-231-FLuc cells were i.v. injected through tail vein into 7-week-old BALB/cJ female mice and NSG female mice, respectively. In vivo metastasis progress was monitored by detecting FLuc signal using IVIS imaging. After metastasis was detected, mice (*n* = 5/group) were treated with saline, cmLumiOpto (10 × 10^10^ ptc/kg-BW mAb-Exo-AAV and 2 mg/kg-BW ViviRen), and cmLumiOpto (same dose) combined with PARPi (50 mg/kg-BW) following the treatment schedule outlined above. TNBC metastasis was assessed once a week using IVIS. Mice were sacrificed when body weight dropped by 20% or other early removal criteria were met. Major organs, including brain, heart, lungs, liver, spleen and kidneys, were harvested for H&E staining, IHC staining, mRNA sequencing, and other post treatment analyses.

### Patient derived xenograft (PDX) model and in vivo treatment

The CD276^+^ TNBC PDX donor mice were obtained from The Jackson Laboratory. Once the volume of donor PDX reached 2,000–3,000 mm^3^, tumors were harvested, minced into 1 × 1 × 1 mm^3^ fragments, and implanted into the mammary fat pad of NSG mice using a 16G needle syringe (or snap frozen and stored in liquid nitrogen). Once the xenografted PDX reached 70–100 mm^3^, mice were divided into two groups (*n* = 4–5) and treated with either saline or cmLumiOpto/PARPi (10 × 10^10^ ptc/kg mAb-Exo-AAV, 2 mg/kg ViviRen, 50 mg/kg PARPi). Tumor size and mouse body weight were monitored twice a week until tumor volume exceeded 1,000 mm^3^ in the control group. Tumor tissue and major organs were then harvested for further post treatment analysis.

### Bulk RNA sequencing

The lung tissues with TNBC metastasis were harvested, dissociated and lysed to extract total mRNA using RNeasy Fibrous Tissue Mini Kit (Qiagen, Germantown, MD, USA). The cDNA library construction and bulk RNA sequencing using Illumina HiSeq™ X Ten platform were carried out at Novogene America (Sacramento, CA, USA). Sequencing reads were mapped to the mouse reference genome (GRCm38) using Hierarchical Indexing for Spliced Alignment of Transcripts version 2 (HISAT2). Differentially expressed genes (DEGs) between treatment and saline groups were identified using edgeR (version 4.2.1) in R. The P-values were adjusted using Benjamini-Hochberg method to control the false discovery rate (FDR), with FDR < 0.05 set as the threshold for DEG selection. Gene Ontology (GO) enrichment analysis was performed using Gene Set Enrichment Analysis (GSEA) method implemented in the clusterProfiler R package. The GO terms with a corrected P-value < 0.05 were considered significantly enriched.

### Paraffin section and Hematoxylin and Eosin (H&E) staining

The harvested tumor tissues and organs were dehydrated in 70% ethanol, cleaned with xylene and coated in paraffin. The paraffin embedded tissue blocks were sectioned at 5 μm thickness using a Leica microtome (Leica Biosystems, Deer Park, IL, USA). The sectioned slides were de-paraffinized with xylene, hydrated with 100 − 70% ethanol, washed with ddH_2_O, and stained with H&E as previously described [23, 31, 40, 51, 52].

### Immunofluorescent staining

TNBC tumor tissue slides were incubated with rabbit anti-TOMM20 polyclonal antibody conjugated to AF488 (Abcam, Cat# ab205486) and anti-cytochrome C antibody-conjugated to AF647 (Biolegend, Cat# 612310) at a dilution of 1:200. The IHC-stained slides were imaged using a Nikon A1R-HD25 confocal microscope (Nikon).

### Statistical analysis

The experimental data were presented as mean ± standard error of the mean (SEM) in this study. Statistical analysis and comparison were performed using two-tailed *t* test and one-way ANOVA followed by post-hoc (Dunnett’s) analysis with GraphPad Prism. *P* < 0.05 was considered statistically significant for all tests.

### Data availability

All raw data generated in this study are available upon request from the corresponding author.

## Results

### CD276 overexpression in TNBCs

**Fig. 1:**
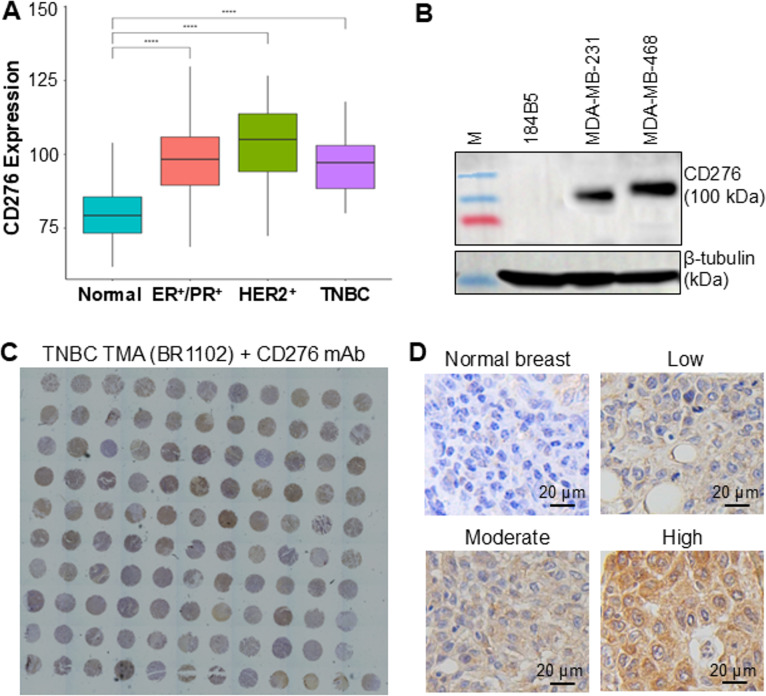
CD276 receptor expression in TNBCs. **A** TCGA dataset analysis of CD276 transcript in ER^+^/PR^+^, HER2^+^ and ER^−^/PR^−^/HER2^−^ breast cancers as compared to normal breast tissue. ****P < 0.0001. **B** Western blotting analyses of surface CD276 expression in human TNBC cell lines and normal breast epithelial cells. **C** IHC staining of TMA of TNBC patients (*n* = 110). **D** Representative IHC images of tissues with minimal, low, medium and high CD276 expressions

The Cancer Genome Atlas (TCGA) dataset analysis revealed that CD276 mRNA levels are significantly higher in TNBC (and other breast cancer) tissues compared to normal breast tissue (Fig. 1A). Western blot analysis confirmed high CD276 expression in TNBC lines MDA-MB-231 and MDA-MB-468, which represent mesenchymal stem-like (MSL), basal-like 2 (BL2), and luminal androgen receptor (LAR) subtypes. In contrast, CD276 expression was minimal in the normal breast epithelial cell line 184B5 (Fig. 1B). IHC staining of TNBC patient TMA (*n* = 110) demonstrated that 23% of cases (25/110) exhibited high CD276 expression, 44% (47/110) had moderate expression, and 33% (35/110) showed minimal or no expression (Fig. 1C). Representative IHC images of normal breast tissue and TNBC cores with varying CD276 expressions levels are shown in Fig. 1D. Collectively, these findings highlight CD276 as a promising target for gene delivery in TNBC.

### CD276 mAb exhibiting high TNBC specificity

To assure the safety to deliver cmLumiOpto gene therapy via targeting CD276 receptor, we analyzed the potential off-target in normal human organs. The IHC staining of normal human tissue using our anti-human/mouse CD276 mAb did not detect significant binding in brain, heart, liver, spleen, lung, kidney, breast and pancreas (Fig S1). Similar IHC staining using mouse normal tissues (liver, kidney, lung, skeletal muscle, brain, heart, stomach, spleen, malignant mouse adrenal gland as positive control) did not detect obvious off-target of our CD276 mAb (Fig. S2).

**Fig. 2:**
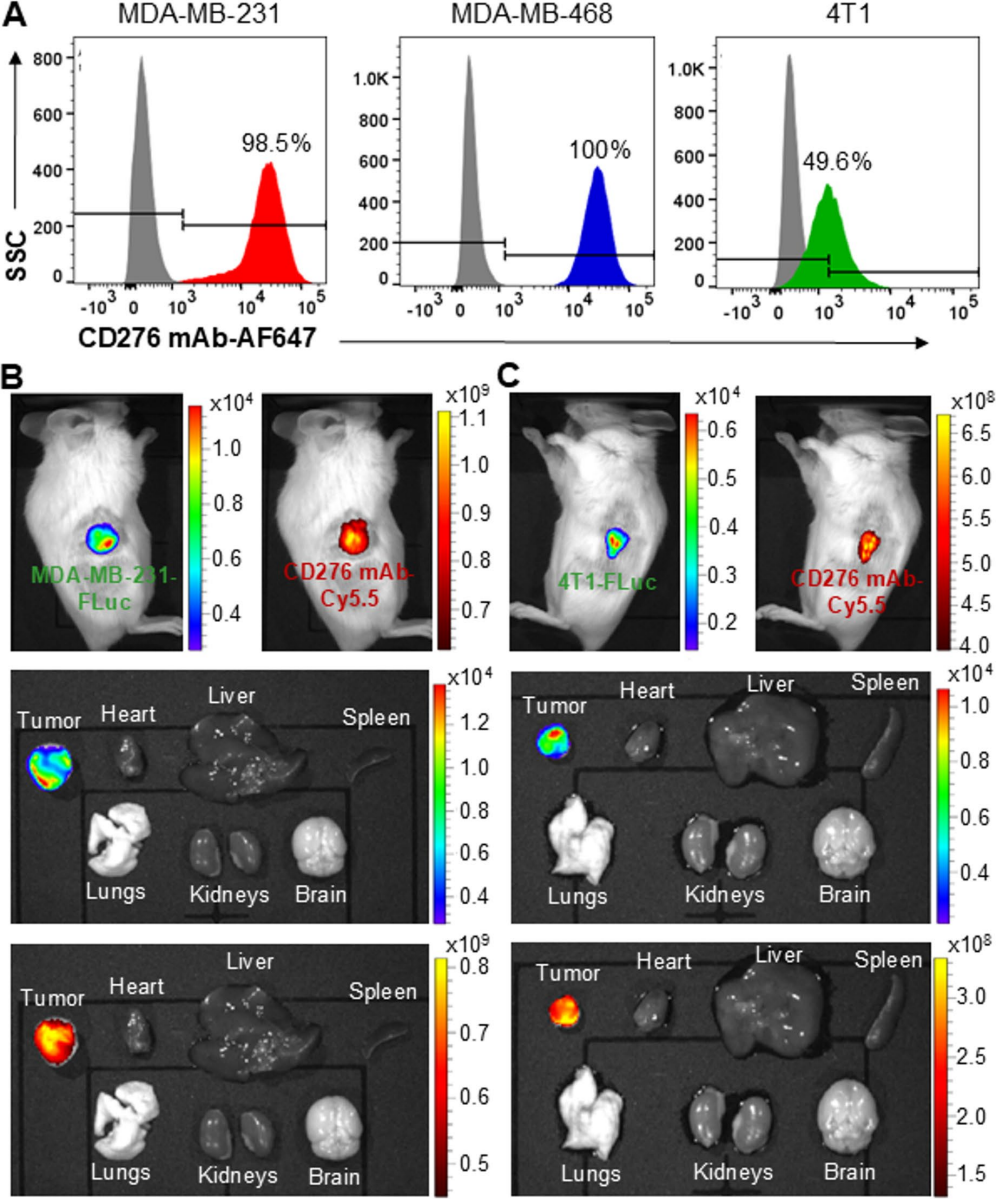
TNBC-targeting of CD276 mAb. **A** Flow cytometry analysis of CD276 surface binding to MDA-MB-231, MDA-MB-468 and 4T1 cells. **B** Representative live-animal and ex vivo IVIS images demonstrating CD276 mAb-Cy5.5 accumulation in human TNBC xenografts 24 h post tail vein injection. **C** Representative IVIS imaging showing CD276 mAb-Cy5.5 accumulation in mouse TNBC xenografts

Our CD276 mAb was produced at large scale in a stirred-tank bioreactor with volumetric titer of ~ 80–120 mg/L from batch bioreactor (Fig. S3A) and subsequently purified using a protein A column (Fig. S3B). The TNBC targeting capability of CD276 mAb was evaluated and confirmed *via* flow cytometry analysis using human MDA-MB-231 and MDA-MB-468 cell lines and mouse 4T1 cell line (Fig. 2A). The surface binding rates were 98.5, 100 and 49.6% in these three lines, respectively, indicating cross-species reactivity of the mAb. Furthermore, the in vivo TNBC targeting ability of CD276 mAb was assessed using NSG mice xenografted with human MDA-MB-231-FLuc (Fig. 2B) and BALB/cJ mice implanted with mouse 4T1-FLuc tumors (Fig. 2C). Live-animal IVIS imaging conducted at 24 h post tail vein injection of 50 µg mAb showed strong overlap of TNBC tumors (FLuc) with CD276 mAb (Cy5.5). Ex vivo IVIS imaging of tumor and major organs, including the heart, liver, spleen, lungs, kidneys and brain, further confirmed the specific targeting of CD276 mAb to TNBC, with no detectable off-targeting accumulation in normal organs. Taken together, both in vitro and in vivo evaluations demonstrated the potential of our CD276 mAb as an effective TNBC-targeting agent for gene therapy delivery.

### Construction and characterizations of CD276 mAb-Exo-AAV

**Fig. 3:**
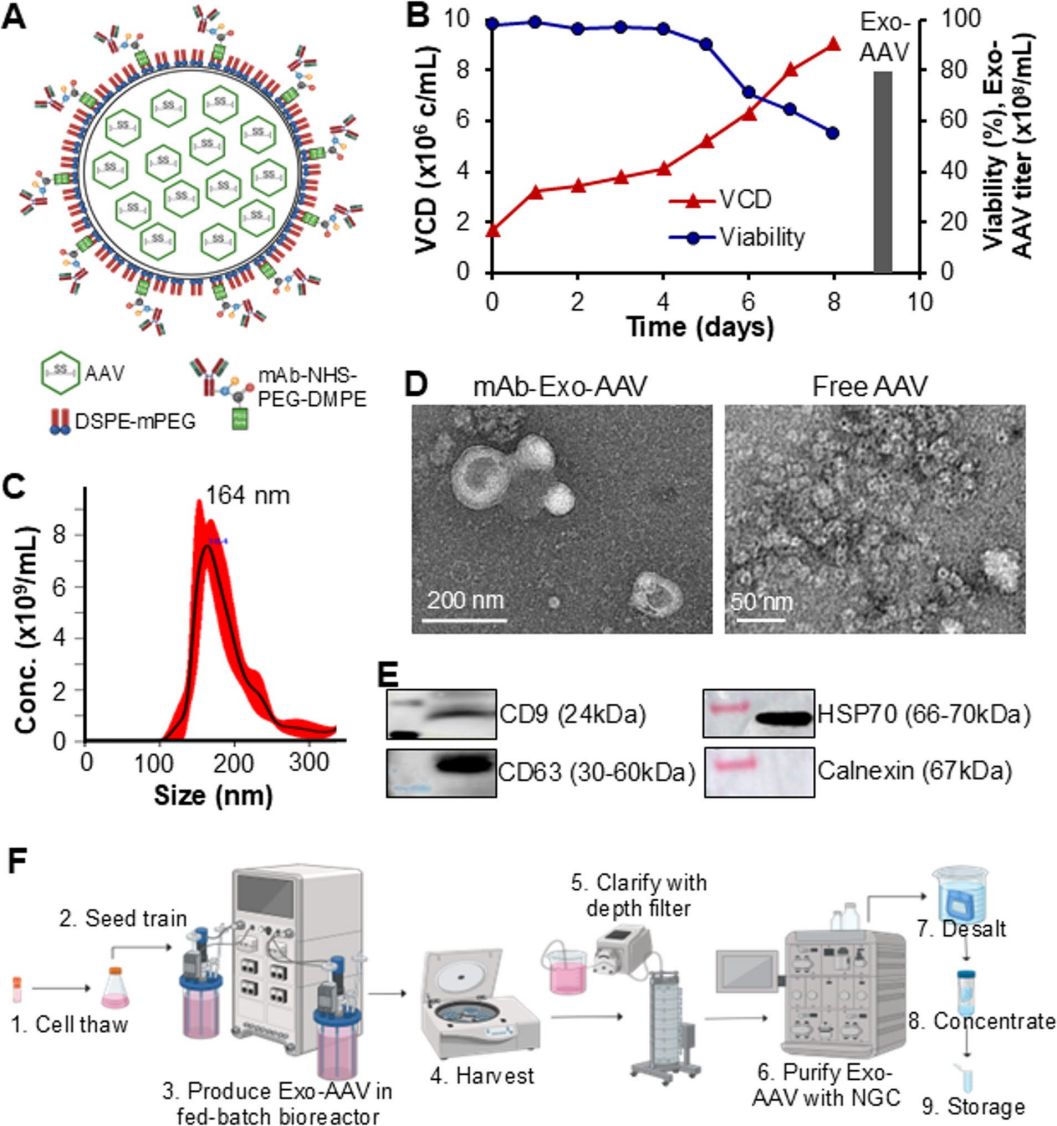
Construction and characterizations of CD276 mAb-Exo-AAV. **A** Structure of mAb-Exo-AAV. **B** Production of Exo-AAV from VPC in 2-L stirred-tank bioreactor at Temp 37 °C, pH 7.0, Agt 210 rpm, and DO 40%. (**C**) NanoSight Pro assay for mAb-Exo-AAV size distribution and titration. **D** TEM images of mAb-Exo-AAV and free AAV. **E** Western blotting analysis confirming exosome biomarkers of CD9, CD63 and HSP70 and negative marker of Calnexin. **F** Process flow diagram of large-scale Exo-AAV biomanufacturing

To achieve TNBC targeting delivery, we packed the cmLumiOpto genes in AAV, harvested and purified Exo-AAV secreted by VPC, and conjugated our CD276 mAb to the surface of exosomes using a DMPE-PEG-NHS linker [23, 51] (Fig. 3A). To enhance circulation stability and reduce renal clearance, the mAb-Exo-AAV was further pegylated with mPEG-DSPE. As illustrated in Fig. 3B, high-yield production of Exo-AAV was achieved in 2-L stirred-tank bioreactor, yielding 9–10 × 10⁹ particles/mL following our optimized protocol [23, 51]. NanoSight Pro analysis revealed a size distribution of 100–300 nm, with an average diameter of 164 ± 25 nm (Fig. 3C). TEM images confirmed the morphology of both Exo-AAV and free AAV particles (Fig. 3D). Western blot analysis detected the presence of key exosome markers CD9, CD63 and HSP70, while the absence of calnexin marker confirmed the high purity of Exo-AAV without endoplasmic reticulum contamination (Fig. 3E).

The production procedure of Exo-AAV was successfully scaled up from 30-mL to 300-mL culture in shaker flask and 2-L culture in bioreactor (Fig. 3F). Key factors influencing Exo-AAV yield and AAV quality included VPC transfection viability, nutrient supplementation, agitation rate, and harvest viability. The purification protocol was scaled up from a 5-mL to a 20-mL size-exclusion chromatography column, followed by ultrafiltration using a 100-kDa regenerated cellulose membrane, which further improved purity while maintaining a 90–95% recovery rate.

In vitro evaluations of cmLumiOpto delivered with CD276 mAb-Exo-AAV

**Fig. 4:**
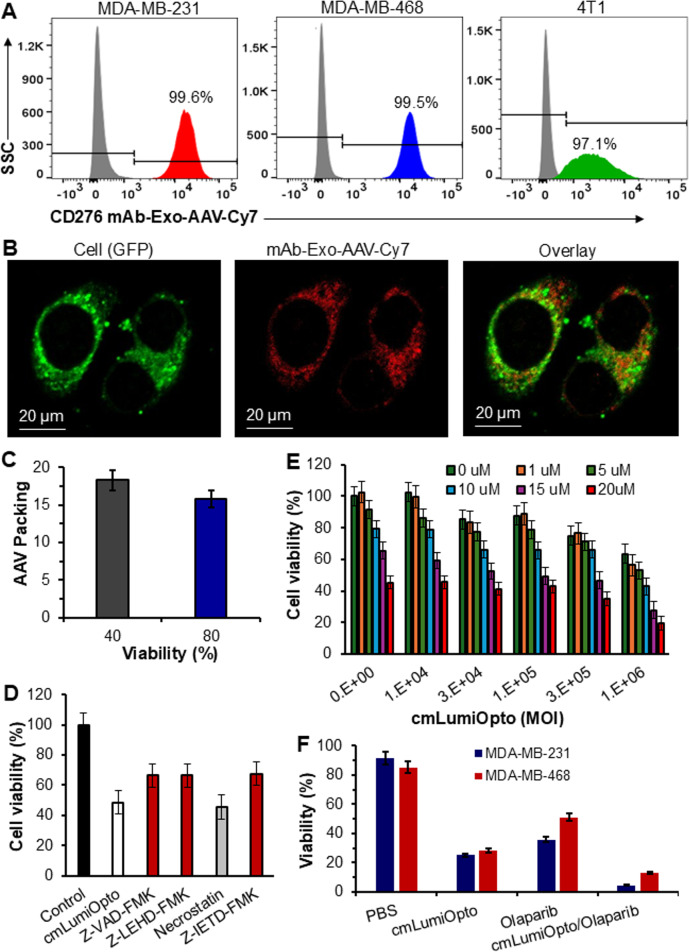
In vitro evaluations of CD276 mAb-Exo-AAV. **A** Flow cytometry analysis of TNBC surface binding of Cy7 labelled mAb-Exo-AAV in MDA-MB-231, MDA-MB-468 and 4T1 cells. **B** Representative confocal images showing internalization of mAb-Exo-AAV-Cy7 (red) in MDA-MB-468 cells (green). **C** Effect of harvest viability on Exo-AAV production yield. **D** Anti-cancer mechanism study using MDA-MB-231 cells treated with saline (control), cmLumiOpto, and cmLumiOpto plus inhibitors of Z-VAD-FMK (pan-caspase inhibitor), Z-LEHD-FMK (caspase-9 inhibitor), necrostatin (necrosis inhibitor) or Z-IETD-FMK (caspase-8 inhibitor). **E** Design of experiment to assess the synergism of cmLumiOpto (0–1,000,000 MOI) and PARPi (0–20 µM) using MDA-MB-231 cells. **F** Cytotoxic effects of cmLumiOpto, PARPi and cmLumiOpto/PARPi in MDA-MB-231 and MDA-MB-468 cells. *n* = 3/group

Flow cytometry analysis revealed high surface binding of CD276 mAb-Exo-AAV labelled with Cy7 in MDA-MB-231 (99.6%), MDA-MB-468 (99.5%) and 4T1 (97.1%) cells (Fig. 4A), confirming strong TNBC-targeting capability. The TNBC transfection and internalization of CD276 mAb-Exo-AAV-Cy7 was validated using confocal microscope in GFP-expressing MDA-MB-468 cells, where the 84% overlay of GFP in cytoplasm and Cy7 signals in the internalized mAb-Exo-AAV indicated high transduction efficiency of the cmLumiOpto gene (Fig. 4B). Functional expression of cmLumiOpto and the surface-bound mAb on Exo-AAV had been confirmed in previous study [23]. While AAV packaging efficiency in Exo-AAV harvested at 40% VPC viability was slightly higher than at 80% viability (18.32 vs. 15.80 gc-AAV/ptc-Exo-AAV, Fig. 4C), we optimized the production by collecting Exo-AAV at 60–80% viability to balance yield and quality. Mechanism analyses further revealed that the apoptosis inhibitors Z-VAD-FMK (pan-caspase), Z-LEHD-FMK (caspase-9) and Z-IETD-FMK (caspase-8) reduced cell death, whereas the necrosis inhibitor necrostatin had no obvious effect (Fig. 4D). These results indicated that cmLumiOpto induces caspase-dependent apoptosis rather than necrosis. To comprehensively evaluate the synergism between cmLumiOpto (0–1 × 10^6^ MOI) and PARPi (0–20 µM), we conducted a design of experiments (DoE). Assessment of MDA-MB-231 cell viability after two days of treatment, analyzed using the Bliss independence model, demonstrated synergistic effects (enhanced cell killing) across most combinations, with the strongest synergy observed at cmLumiOpto concentrations >1 × 10^5^ MOI and PARPi concentrations >10 µM (Fig. 4E). The cytotoxic effect of cmLumiOpto delivered with mAb-Exo-AAV, Olaparib, and combination were evaluated in MDA-MB-231 and MDA-MB-468. Cell viability was significantly reduced to 25.03–28.22%, 35.86–51.17%, and 4.47–13.32%, respectively, following treatment (Fig. 4F). Notably, cmLumiOpto gene therapy demonstrated higher cytotoxicity than Olaparib alone, and their combination further enhanced therapeutic potency against TNBC cells (Fig. 4F). These results showed that CD276 mAb-Exo-AAV is a promising gene delivery system, with high TNBC selectivity, transduction efficiency, and synergistic therapeutic potential when combined with PARP inhibition.

### Evaluation of anti-TNBC efficacy in immunocompromised models

**Fig. 5:**
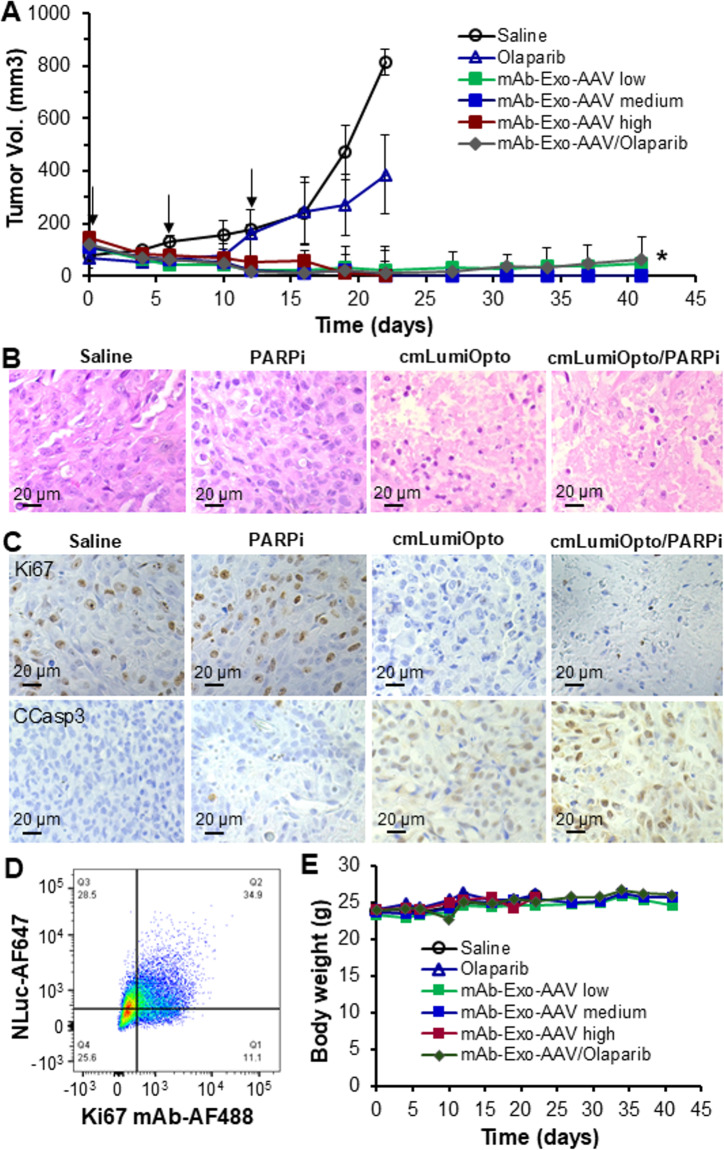
Evaluation of anti-TNBC efficacy in MDA-MB-231 xenografted NSG mouse models. **A** Tumor volume profiles treated with i.v. administration of cmLumiOpto at doses of 2 × 10^10^ ptc/kg (low), 10 × 10^10^ ptc/kg (medium) or 30 × 10^10^ ptc/kg (high) following Q7Dx3 as indicated by arrow, PARPi at dose of 50 mg/kg, cmLumiOpto (medium dose, 10 × 10^10^ ptc/kg-BW) in combination with Olaparib (50 mg/kg), and saline (control). Data were presented as mean ± SEM, *n* = 6. *P < 0.01 vs. saline using ANOVA followed by Dunnett’s *t*-test. **B** H&E staining of tumor tissues harvested on Day 19. Scale bar equals 20 μm. **C** IHC stained tumor tissues with Ki67 (proliferation marker) antibody and CCasp3 antibody (apoptosis marker). Scale bar equals 20 μm. **D** Flow cytometry analysis of dissociated MDA-MB-231 xenograft. *n* = 4.1 × 10^6^ cells were co-stained with 1 µg of AF488 labelled Ki67 antibody and 1 µg of AF647 labelled NLuc antibody. **E** Body weight profiles

The anti-cancer efficacy of cmLumiOpto at varying doses (low: 2, medium: 10 and high: 30 × 10^10^ ptc/kg-BW), PARPi monotherapy, and a combination of low dose cmLumiOpto with PARPi was evaluated in MDA-MB-231 xenografted NSG mouse models (*n* = 6/group). Following the first injection, tumor volume in groups receiving cmLumiOpto alone or in combination with PARPi decreased from 107 to 145 mm^3^ to 42–78 mm^3^ within one week (Fig. 5A). Medium and high doses of cmLumiOpto induced further tumor shrinkage (20–50 mm^3^) after the second injection (week 2) and achieved complete tumor regression after the 3rd injection (week 3), with no recurrence observed through weeks 4–6. In contrast, tumors progressed to 815 mm³ in control group (saline) and 386 mm³ in PARPi monotherapy group, respectively, by week 3, necessitating early sacrifice due to ulceration (> 2 mm) before reaching 1,000 mm³. It should be noted that low-dose cmLumiOpto alone or cmLumiOpto/PARPi combination experienced minor tumor size rebound after treatment cessation, with volumes reaching 15–31 mm³ (week 4), 34–35 mm³ (week 5), and 48–61 mm³ (week 6).

To assess whether the observed tumor size increase in low dose cmLumiOpto and cmLumiOpto/PARPi groups resulted from recurrence, the harvested tumor tissues were evaluated with histological analysis. H&E staining revealed severe tumor cell death and reduced cancer cell density in the cmLumiOpto group, while the cmLumiOpto/PARPi group exhibited TME disruption characterized by the fluid-like tissue (Fig. 5B). These findings suggested that the slight increase of tumor volume between Days 22–41 was unlikely due to TNBC recurrence but may involve non-cellular factors. Further investigation is warranted to determine potential contributions from inflammatory responses, necrotic core expansion, vascular remodeling, and stromal changes. IHC staining showed that the percentage of Ki67-positive cells (a proliferation marker) ranged from 12 to 58% in the saline and PARPi groups, compared to 2–6% in the cmLumiOpto and cmLumiOpto/PARPi groups. Similarly, cleaved caspase-3-positive cells (an apoptosis marker) were observed at 3–8% in the saline and PARPi groups, versus 25–55% in the cmLumiOpto and cmLumiOpto/PARPi groups. These results confirmed significant apoptotic activity and proliferation inhibition in treatment groups (Fig. 5C). While PARPi monotherapy slowed tumor progression, H&E and IHC staining revealed no significant tumor cell death or growth inhibition (Figs. 5B-C), emphasizing the superior therapeutic efficacy of combining gene therapy with chemotherapy through synergistic anti-cancer effects.

To further evaluate in vivo infection efficiency, fresh tumor tissues were analyzed on Day 5 post-injection. Flow cytometry of dissociated tumor cells stained for NLuc and Ki67 revealed that ~ 75.9% of TNBC cells were successfully infected with CD276 mAb-Exo-AAV and expressed functional cmLumiOpto gene. These results suggested that multiple administrations and chemotherapy integration may be required to enhance therapeutic efficacy (Figs. 5A-B).

Importantly, no significant body weight changes were observed across treatment groups (Fig. 5E), indicating minimal systemic toxicity. Furthermore, H&E staining of major organs (brain, heart, lungs, liver, spleen, kidneys) showed no signs of inflammation, apoptosis, or necrosis (Fig. S4), confirming the safety profile of cmLumiOpto and cmLumiOpto/PARPi therapy at the tested doses.

### Evaluation of anti-TNBC efficacy in distant metastatic models

**Fig. 6:**
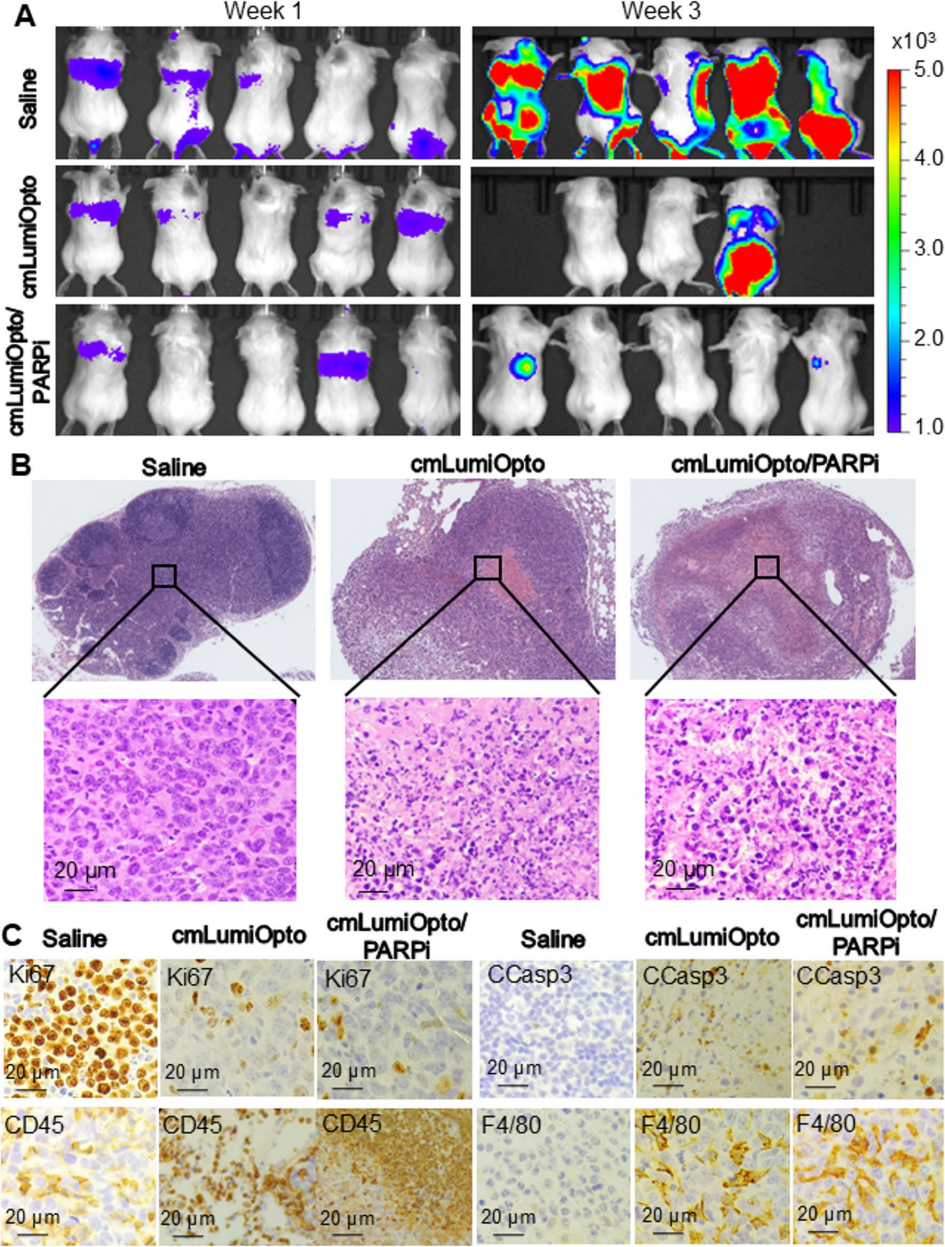
Assessment of anti-TNBC efficacy in 4T1 metastatic models. Mice were treated with cmLumiOpto (medium dose 10 × 10^10^ ptc/kg, i.v. injection on Days 0 and 7)/Olaparib (50 mg/kg, oral administration) and saline (control). *n* = 5. **A** IVIS imaging of BALB/cJ mice carrying metastatic 4T1-FLuc. **B** H&E staining of lung tissues with TNBC metastasis. Scale bar equals 20 μm. **C** IHC staining of tumors spot treatment using markers of cell proliferation (Ki67), apoptosis (CCasp3), activated T/NK (CD45), and macrophage cells (F4/80). Scale bar equals 20 μm

*Metastasis inhibition in immunocompetent models.* Following the detection of TNBC (4T1-FLuc) metastasis *via* IVIS imaging, immunocompetent mouse models (*n* = 5/group) were treated with saline, cmLumiOpto (10 × 10^10^ ptc/kg-BW) and cmLumiOpto/PARPi (10 × 10^10^ ptc/kg-BW, 50 mg/kg-BW) *via* i.v. injection or drinking water supplementation. PARPi monotherapy was not included due to its limited efficacy in primary tumor models (Fig. 5). By week 3, IVIS imaging revealed extensive cancer metastasis in the saline group, moderate metastasis reduction in the cmLumiOpto group (two mice were lost before final imaging due to an accident), and complete blockage or elimination of metastases in the combination treatment group (Fig. 6A). Lung tissues with extensive TNBC metastasis were collected for histological and mechanism of action (MOA) analyses. Whole-slide scanning revealed dense tumor colonies in the lung tissue of saline-treated mice (Fig. 6B), whereas treatment with cmLumiOpto or cmLumiOpto/PARPi led to tumor necrosis, despite the presence of metastatic lesions. Microscopic imaging further highlighted remarkable differences between groups, with widespread tumor cell death and necrosis in treated mice, while tumors in saline-treated controls remained intact and proliferative (Fig. 6B).

IHC staining of tumor tissues (Fig. 6C) demonstrated significant suppression of prolieration marker Ki67 and an increase in apoptosis marker cleaved caspase-3 in treatment groups. Specifically, the precentage of Ki67-positive cells were 85%, 45% and 15%, while those of CCasp3-positive cells were < 5%, 40% and 40% in the saline, cmLumiOpto, and cmLumiOpto/PARPi groups, respectively. These data confirmed the ability of our therapies to induce cancer cell death and inhibit metastatic proliferation. Additionally, tumor-infiltrating NK cells (CD45^+^) were enriched from < 5% in the saline group to 35% and 75% in the cmLumiOpto and cmLumiOpto/PARPi treatment groups, respectively. Macrophage phagocytosis (F4/80^+^)-positive cells were also enhanced from < 5% in the saline group to 45% and 55% in the cmLumiOpto and cmLumiOpto/PARPi treatment groups, respectively. This histological analysis suggests an immune-mediated tumor clearance mechanism. Furthermore, IHC staining of lung tissues harboring metastatic TNBC was performed with antibodies against CD8 (T cells), NK1.1 (NK cells) and NKp46 (NK cells). The percentages of positive cells were as follows: CD8, 2% and 65%; NK1.1, 5% and 75%; and NKp46, 1% and 55% in the saline and cmLumiOpto/PARPi groups, respectively (Fig. S5A). This data further confirmed immune cell infiltration in tumor microenvironment by cmLumiOpto treament. These findings indicated that tumor immune regulation contributed to the therapeutic effects, which needs further investigation through mechanism-of-action (MOA) studies.

Importantly, no significant changes in body weight were observed across groups (Fig. S5A), and H&E staining of major organs (brain, heart, liver, spleen, kidneys) detected no toxicity or tissue damage in treatment groups (Fig. S5B), consistent with the safety profile observed in primary TNBC models (Fig. S4).

*Metastasis inhibition in immunocompromised models.* To further validate the anti-metastatic efficacy of cmLumiOpto/PARPi, a similar study was conducted in a second mouse model using immunocompromised NSG female mice bearing metastatic human TNBC (MDA-MB-231-FLuc). IVIS imaging revealed that cmLumiOpto/PARPi treatment significantly reduced TNBC metastases (Fig. S6A). Consistent with the immunocompetent model, therapeutic administration did not impact mouse body weight (Fig. S6B), and H&E staining of lung tissues demonstrated lower metastatic burden three weeks post-treatment (Fig. S6C). These data further confirmed the efficacy of cmLumiOpto/PARPi in suppressing TNBC metastasis across distinct preclinical models.

### Anti-cancer mechanisms

**Fig. 7:**
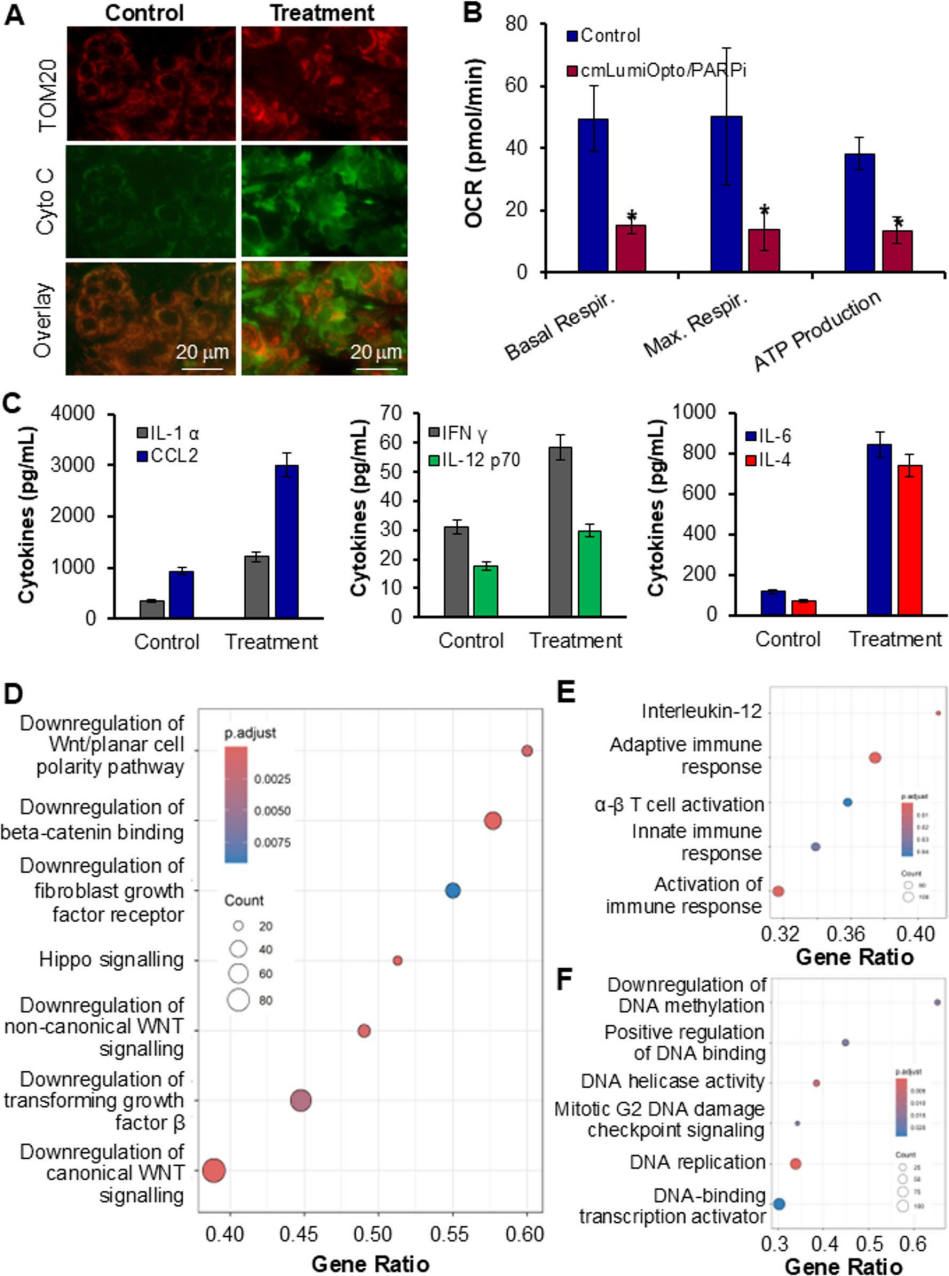
Investigation of anti-cancer mechanisms. **A** Immunofluorescence assay of cytochrome C release in 4T1 xenografts. **B** Seahorse assay to analyze the mitochondrial function in MDA-MB-231 post treatment. *n* = 4. **C** Luminex assay analysis of tumour tissues with 4T1 metastasis. **D** RNA-seq demonstrating cmLumiOpto downregulated metastasis signaling of Wnt, TGF-β, IL-6 and FGFR. **E** Upregulation of tumor immunity (T cells) and IL12. **F** Downregulation of DNA damage response and histone lysine methylation

To elucidate the anti-cancer mechanism of action (MOA) of combined cmLumiOpto/PARPi, we first investigated its impact on TNBC mitochondrial structure and function. Immunofluorescence staining of TNBC tumors with TOM20 (outer mitochondrial membrane marker) and cytochrome c antibodies revealed intact mitochondrial architecture in untreated controls, with co-localized TOM20 and cytochrome c (Fig. 7A, left). In contrast, treated samples exhibited >66% of cytochrome c release from the mitochondria, indicative of mitochondrial injury and collapse (Fig. 7A, right), consistent with our previous findings on cmLumiOpto monotherapy [31]. Mitochondrial functional impairment was quantified using the Seahorse assay in MDA-MB-231 cells treated with cmLumiOpto/PARPi for 48 h (70–80% viability; Fig. 7B). Oxygen consumption rate (OCR) analysis demonstrated significant reductions in basal respiration (49.4 to 15.2 pmol/min), maximal respiration (50.4 to 14.0 pmol/min), and ATP production (38.2 to 13.6 pmol/min). These data revealed severe mitochondrial bioenergetic disrupption, leading to mitochondria dysfunction and irreversable cancer cell death, by the targeted delviered cmLumiOpto.

Beyond the immunohistochemical analysis of immune function and immune cells infiltration (Fig. 6C), we harvested and dissociated the lung tissues harboring TNBC (4T1) metastases. The extract was applied to perform a multiplex Luminex assay to quantify cytokines secretion within the TME (Fig. 7C). It is observed that the cmLumiOpto/PARPi markedly upregulated several key cytokines, including IL-1α (2.41-fold; 355 to 1,211 pg/mL) and CCL2 (2.24-fold; 927 to 3,008 pg/mL). Additionally, IFN-γ (1.87-fold; 31 to 58 pg/mL) and IL-12 p70 (1.67-fold; 17 to 29 pg/mL) were elevated, while IL-6 (7.18-fold; 117 to 843 pg/mL) and IL-4 (10.47-fold; 70 to 739 pg/mL) exhibited striking increases. In this study, the secretion of anti-tumor cytokines IFN-γ and IL-12 and the dual-role cytokines IL-1α, IL-6 and IL-4 was significantly increased by the cmLumiOpto/PARPi combination. The higher level of pro-tumor CCL2 was also observed in treatment group, which need further investigation in future. These data, together with the detected immune cells activation and infiltration (Figs. 6C) in tumor microenvironment, suggested immunomodulatory response, which could benefit anti-tumor efficacy of gene therapy-chemotherapy.

Bulk RNA-Seq analysis of lung tissues provided further insights into TNBC metastasis regulation. Differential gene expression (DGE) analysis revealed suppression of multiple metastasis-associated pathways by cmLumiOpto/PARPi (Fig. 7D). Canonical and non-canonical Wnt signaling, key drivers of epithelial-mesenchymal transition, invasion, angiogenesis and colonization, were inhibited. Downregulation of fibroblast growth factor receptor (FGFR) signaling curtailed downstream RAS/MAPK and PI3K/AKT activation, while reduced transforming growth factor β (TGF-β) expression correlated with diminished metastatic potential. The observation of metastasis reduction in two mouse models (Figs. 6 and S6) and the downregulation of these metastatic signaling pathways indicated that targeting cancer mitochondria can effectively inhibit, reduce or eliminate the metastasis of aggressive cancers. We need to further investigate the mechanism and validate this finding by using new two-direction research approaches to depolarize and hyperpolarize mitochondria in future study. In addition, the Hippo signaling, a tumor suppressor, was upregulated by treatment. In the TME, immune activation was evident through upregulated immune response and enhanced αβ T-cell activity and IL-12 production (Fig. 7E). These results were consistent with the observed immunity upregulation and immune cells infiltration in tumor microenvironment (Figs. 6C and 7C). It indicated that the immune function caused by CD276 mAb-Exo-AAC could offer additional therapeutic benefits in targeted cancer treatment. Finally, DNA damage responses, methylation, and helicase activity, known to restrict proliferation and metastasis, were significantly reduced (Fig. 7F). Our previous study [23] showed that cancer mitochondria depolarization and mitochondrial inner membrane potential collapse cause persistent DNA damage. The FDA approved PARPi can disrupt the repair of DNA damage in breast cancers. This RNA-Seq data underscored the multifaceted anti-metastatic impact of the combination of cmLumiOpto/PARPi.

### Efficacy of cmLumiOpto/PARPi in PDX models

**Fig. 8:**
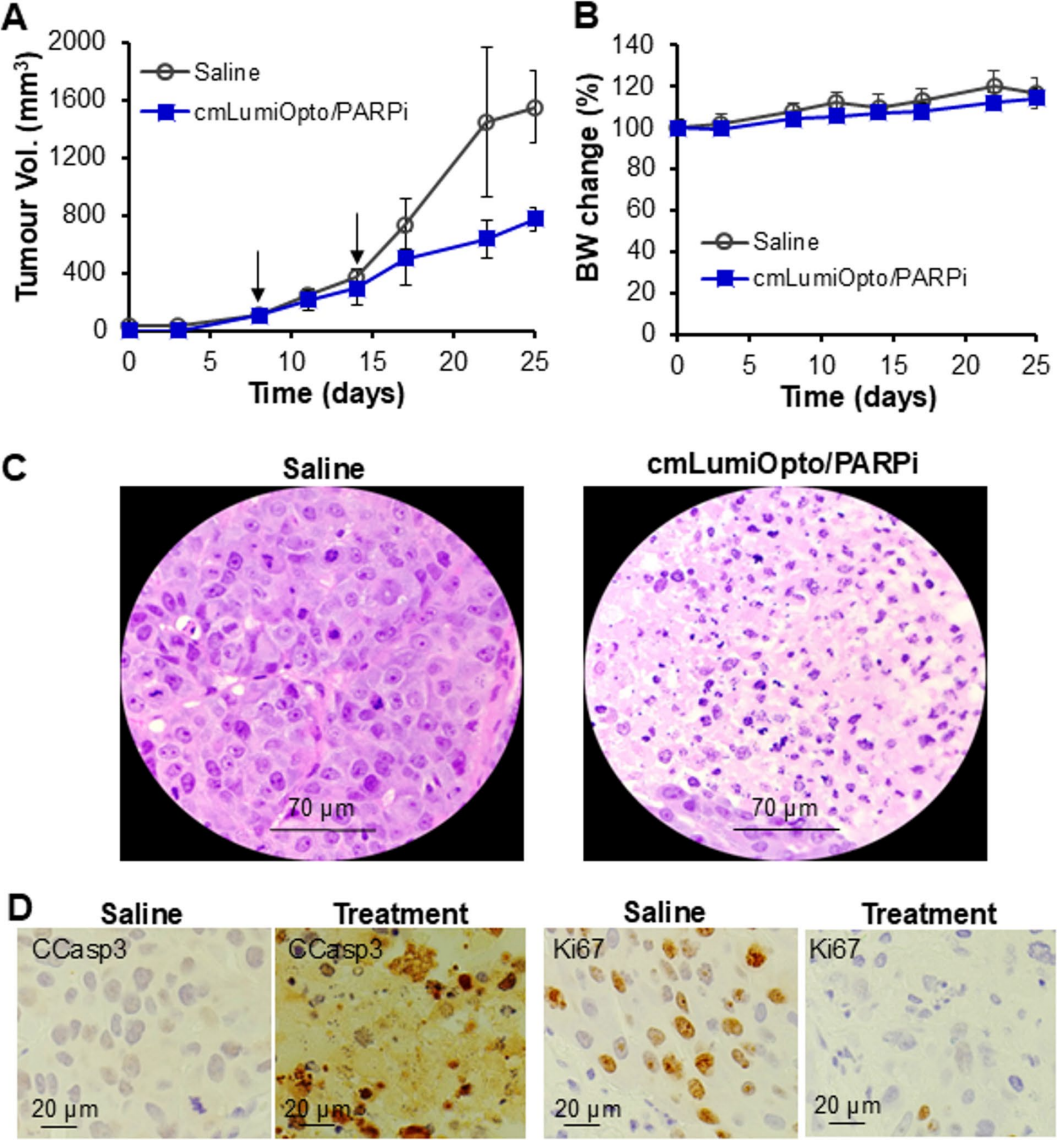
Test of anti-TNBC efficacy in PDX models. Mice were treated with cmLumiOpto (medium dose)/PARPi (50 mg/kg) and saline (control). *n* = 4–5. **A** Tumor volume profiles following treatment as indicated with arrow. *P < 0.001 vs. saline using ANOVA followed by Dunnett’s *t*-test. **B** Body weight profiles. **C** H&E staining of tumor tissue post treatment to confirm cell death. **D** IHC stained tumor tissues with Ki67 (proliferation marker) antibody and CCasp3 antibody (apoptosis marker)

To evaluate the therapeutic efficacy of cmLumiOpto/PARPi in a clinically relevant setting, PDX xenografted mouse models were established to preserve the heterogeneity and TME of human TNBC. When tumors reached ~ 100 mm^3^, mice received two administrations of cmLumiOpto/PARPi (10 × 10^10^ ptc/kg-BW, 50 mg/kg-BW), as indicated by arrows (Fig. 8A). By Day 25, tumor volume in the treatment group was 50% lower than in the saline group (776 vs. 1,551 mm^3^). Body weight profiles showed no significant difference between treatment and control groups (Fig. 8B), and H&E staining of major organs (brain, heart, lungs, liver, spleen, kidneys) revealed no signs of inflammation or necrosis (Fig. S7). In addition, five doses (2 × 10^10^, 5 × 10^10^, 10 × 10^10^, 20 × 10^10^ and 50 × 10^10^ ptc/kg BW) of cmLumiOpto combined with 50 mg/kg of Olaparib (PARPi) were administered to BALB/cJ mice via tail vein (*n* = 2), with saline serving as the control. Blood samples were collected two weeks after treatment for complete blood count analysis (Fig. S8), which demonstrated minimal hematological toxicity. Taken together, the cancer mitochondrial-targeted cmLumiOpto in combination with PARPi does not cause toxicity at the tested doses. Histological analysis of tumor tissues demonstrated widespread cell death within the TME following cmLumiOpto/PARPi treatment, while tumors in the saline group remained intact (Fig. 8C). Furthermore, IHC staining revealed that the combined gene-chemotherapy induced significant apoptosis and proliferation inhibition (Fig. 8D), with CCasp3-positive cells at < 5% and > 50% and Ki67 positive cells at 70% and < 5%, in saline control and cmLumiOpto/PARPi treatment group, respectively, underscoring its potential to effectively suppress heterogeneous TNBC tumors in PDX models.

## Discussion

Conventional chemotherapeutics for TNBC is hampered by low response rates and synthetic lethality, prompting the need for more effective strategies. To address this challenge, we developed a novel cancer mitochondria-targeting luminoptogenetics system (cmLumiOpto) that induces severe mitochondrial dysfunction, DNA damage and subsequent cell death. Leveraging our prior development of CD276 mAb for drug delivery to TNBC cells [31], we engineered mAb-Exo-AAV construct to deliver cmLumiOpto gene specifically to TNBC cells in vivo. This strategy selectively targets TNBC cells, depolarizes cancer mitochondria, and triggers severe mitochondrial dysfunction, culminating in robust tumor cell death and collapse of the TME without detectable off-target toxicity. We comprehensively evaluated cmLumiOpto as a monotherapy and in combination with PARPi across four TNBC animal models, demonstrating elimination of primary tumors and significant suppression or complete inhibition of metastasis when combined with PARPi.

The cmLumiOpto/PARPi combination represents a synergistic, translatable therapeutic paradigm with distinct advantages over traditional TNBC treatments such as chemotherapy [1, 2] and gene therapy (e.g., p53 [56, 57]). First, cmLumiOpto harnesses sustained intracellular bioluminescence from Nanoluciferase to activate light-gated mitochondrial rhodopsin channels, directly collapsing the DY_m_ and triggering DNA damage. Unlike subtype-specific therapies reliant on endogenous signaling, which are often disrupted in TNBC, cmLumiOpto directly targets mitochondrial function, reducing the likelihood of resistance in heterogeneous tumors. Second, PARPi exploits cmLumiOpto-induced DNA repair deficiencies, amplifying cancer cell lethality. This dual mechanism integrates direct cell death, apoptosis, and autophagy (*via* cmLumiOpto) with DNA damage and metabolic suppression (*via* mitochondrial depolarization and PARPi), effectively targeting metastatic TNBC cells. In immunocompetent models, this synergy eradicated tumors and activated tumoral immunity, partly through CD276 mAb-mediated neutralization of immunosuppressive signals [25–27, 58, 59], enhancing T/NK cell infiltration.

Mitochondria are pivotal regulators of cancer metastasis, modulating ATP production, reactive oxygen species and signaling pathways critical for proliferation, genome stability, and immune evasion [60, 61]. Alterations in mitochondrial genetics and metabolism underpin metastatic cascades [62–64], while mitochondrial stress responses confer resistance to cytotoxic therapies [65–69]. Here, cmLumiOpto-induced mitochondrial collapse repressed metastasis-related signaling, including proliferation, vascular invasion, and TME modulation, as confirmed by bulk RNA sequencing in distant metastatic mouse models, where lung metastasis was inhibited or eliminated. This underscores the potential of mitochondria-targeted therapies to manage metastatic TNBC.

AAV is a promising delivery vehicle of therapeutic genes [70] due to its advantages, including long-term transgene expression, high stability and ability to infect a broad range of cell types. Eight AAV-based therapies, such as Luxturna, Roctavian and Zolgensma, have been approved to date, and approximately 300 clinical trials for treating Alzheimer, Parkinson and other diseases are on-going [71–74]. Despite these achievements, several major clinical challenges limit their applications, including pre-existing immunity from AAV-neutralizing antibodies, immunogenicity, and high-dose-induced hepatotoxicity, neurotoxicity and cardiotoxicity [75, 76]. In addition to AAV, FDA also approved lentivirus vectors for ex vivo genetic modification of cells, such as Zynteglo, Skysona and Abecma, for treating lymphoma, myeloma and rare diseases. The lack of targeted delivery has limited the in vivo application especially for cancer treatment. Unlike AAV and lentivirus, our mAb-Exo-AAV provides an effective targeted delivery vehicle for therapeutic genes, enabling gene therapy to treat low-grade, advanced and metastatic cancers. Our vehicle comprises a cancer-selective *cfos* promoter, TNBC-targeting anti-CD276 mAb and the packed AAV carrying the cmLumiOpto genes. Compared to free AAV, mAb-Exo-AAV enables targeted delivery, high circulation stability, repeated dosing, and escape from AAV neutralization [77–80]. CD276, overexpressed in 67% of patients across subtypes, is an ideal target for primary and metastatic TNBC. Our CD276 mAb not only facilitates delivery but also restores effector immune functions in the TME by neutralizing inhibitory signaling [31]. Importantly, the CD276 mAb exhibits favorable drug delivery parameters and plasma stability. TME modulation is characterized by the increased T/NK cell infiltration and activation [31]. Therefore, the mAb-Exo-AAV not only targets cancer but also enhances tumoral immunity. Furthermore, the Exo-AAV platform supports conjugation with additional mAbs (e.g., targeting EGFR [81–85], Trop-2 [12–14, 86, 87], NK-1R [88] or MET [89–91]), broadening its applicability to heterogeneous TNBCs and potentially other cancers, such as non-small cell lung cancer. The established biomanufacturing process for mAb-Exo-AAV, utilizing stirred-tank bioreactor and liquid chromatography, enables large-scale production [40, 51].

In summary, this study establishes the proof-of-concept for a novel combinatorial gene-chemotherapy approach using mitochondria-targeted cmLumiOpto in concert with PARPi. The integration of direct mitochondrial disruption, enhanced DNA damage, and immune activation offers a compelling strategy for overcoming the challenges posed by heterogeneous and metastatic TNBC. Future studies will optimize dosing schedules and explore dual-mAb targeting in clinically relevant models (e.g., PDX and humanized mice) to enhance the treatment efficacy and translational potential. Comprehensive toxicology and biodistribution analyses will further support preclinical development toward investigational new drug (IND) applications and clinical trials.

## Supplementary Information

Supplementary Material 1. Figure S1. Evaluation of potential off-target of our CD276 mAb.Representative IHC images of human normal organs stained with our humanized CD276 mAb. Scale bar equals 70 µm. Figure S2. Evaluation of CD276 expression and tumor-specificity of our CD276 mAb in mouse.Representative IHC images of mouse normal organs stained with our humanized anti-human/mouse CD276 mAb. Mouse malignant mouse adrenal gland was used as control. *n*=3. Figure S3. Production of humanized CD276 mAb. (A) Production of mAb in 2-L stirred-tank bioreactor at Temp 37^o^C, Agt 140 rpm, DO 40%, and pH 7.0. (B) Purification using liquid chromatography with Bio-Scale Mini UNOsphere SUPrA affinity column. Loading buffer A: 0.02 M Na_3_PO_4_, 0.02 M Na_3_C_6_H_5_O_7_, pH 7.5. Elution buffer B: 0.1 M NaCl, 0.02 M Na_3_C_6_H_5_O_7_, pH 3.0. Figure S4. H&E staining of major organs harvested from MDA-MB-231 xenografted NSG mouse models, including brain, heart, lungs, liver, spleen and kidneys. Figure S5. Toxicity assessment in 4T1 metastasis models. (A) IHC staining of lung tissues carrying TNBC metastasis. (B) Body weight profiles. (C) H&E staining of major organs harvested from 4T1 metastatic models, including brain, heart, lungs, liver, spleen and kidneys. Scale bar equals 20 µm. Figure S6. Anti-TNBC efficacy in MDA-MB-231 metastatic models. (A) IVIS imaging of NSG carrying TNBC metastasis. *n*= 5. Mice were treated with ccmLumiOpto (medium dose, 10x10^10^ptc/kg-BW)/Olaparib (50 mg/kg), and saline (control). (B) Body weight changes. (C) H&E staining of lung tissues with TNBC metastasis. Figure S7. H&E staining of major organs harvested from PDX models, including brain, heart, lungs, liver, spleen and kidneys. Scale bar equals 20 µm. Figure S8. Complete blood cell count demonstrated low peripheral toxicity of cmLumiOpto/PARPi in BALB/cJ mice. n=2

## Data Availability

All raw data generated in this study are available upon request from the corresponding author.All data generated or analyzed during this study are included in the main text or the supplementary materials of this article. The bulk RNA-seq data generated in this study are publicly available in Gene Expression Omnibus (GEO) at accession number GSE305990. The transcript data of CD276 in TNBC in this study were obtained from GEO repository dataset. No datasets were generated or analyzed during the current study. Most materials except the anti-CD276 mAb and Exo-AAV used in the study are commercially available. The NSG (NOD.Cg-Prkdcscid Il2rgtm1Wjl/SzJ) and BALB/cJ mice were purchased from Jackson Laboratory.

## References

[1] Liedtke C, Mazouni C, Hess KR, Andre F, Tordai A, Mejia JA, et al. Response to neoadjuvant therapy and long-term survival in patients with triple-negative breast cancer. J Clin Oncol. 2008;26(8):1275–81. 10.1200/JCO.2007.14.4147. 18250347 10.1200/JCO.2007.14.4147

[2] Silver DP, Richardson AL, Eklund AC, Wang ZC, Szallasi Z, Li Q, et al. Efficacy of neoadjuvant cisplatin in triple-negative breast cancer. J Clin Oncol. 2010;28(7):1145–53. 10.1200/JCO.2009.22.4725. 20100965 10.1200/JCO.2009.22.4725 PMC2834466

[3] Nedeljkovic M, Damjanovic A. Mechanisms of chemotherapy resistance in Triple-Negative breast cancer-How we can rise to the challenge. Cells. 2019;8(9). 10.3390/cells8090957. Epub 2019/08/25. 10.3390/cells8090957 PMC6770896 31443516

[4] Wein L, Loi S. Mechanisms of resistance of chemotherapy in early-stage triple negative breast cancer (TNBC). Breast. 2017;34(Suppl 1):S27–30. 10.1016/j.breast.2017.06.023. Epub 2017/07/03. 28668293 10.1016/j.breast.2017.06.023

[5] Martinelli E, De Palma R, Orditura M, De Vita F, Ciardiello F. Anti-epidermal growth factor receptor monoclonal antibodies in cancer therapy. Clin Exp Immunol. 2009;158(1):1–9. 10.1111/j.1365-2249.2009.03992.x. 19737224 10.1111/j.1365-2249.2009.03992.x PMC2759052

[6] Flynn JF, Wong C, Wu JM. Anti-EGFR therapy: mechanism and advances in clinical efficacy in breast cancer. J Oncol. 2009;2009:526963. 10.1155/2009/526963. 19390622 10.1155/2009/526963 PMC2668926

[7] Cheung A, Opzoomer J, Ilieva KM, Gazinska P, Hoffmann RM, Mirza H, et al. Anti-folate receptor alpha-directed antibody therapies restrict the growth of triple-negative breast cancer. Clin Cancer Res. 2018;24(20):5098–111. 10.1158/1078-0432.CCR-18-0652. 30068707 10.1158/1078-0432.CCR-18-0652 PMC6193548

[8] Frontera ED, Khansa RM, Schalk DL, Leakan LE, Guerin-Edbauer TJ, Ratnam M, et al. IgA Fc-folate conjugate activates and recruits neutrophils to directly target triple-negative breast cancer cells. Breast Cancer Res Treat. 2018;172(3):551–60. 10.1007/s10549-018-4941-5. 30155754 10.1007/s10549-018-4941-5 PMC6235697

[9] Romero D. Benefit in patients with PD-L1-positive TNBC. Nat Rev Clin Oncol. 2019;16(1):6. 10.1038/s41571-018-0127-7. 30401935 10.1038/s41571-018-0127-7

[10] Marra A, Viale G, Curigliano G. Recent advances in triple negative breast cancer: the immunotherapy era. BMC Med. 2019;17(1):90. 10.1186/s12916-019-1326-5. 31068190 10.1186/s12916-019-1326-5 PMC6507064

[11] Zhu X, Zhang Q, Wang D, Liu C, Han B, Yang JM. Expression of PD-L1 attenuates the positive impacts of High-level Tumor-infiltrating lymphocytes on prognosis of Triple-negative breast cancer. Cancer Biol Ther. 2019;20(8):1105–12. 1595282. PubMed PMID: 30929569; PMCID: PMC6605986. 30929569 10.1080/15384047.2019.1595282 PMC6605986

[12] Bardia A, Hurvitz SA, Tolaney SM, Loirat D, Punie K, Oliveira M, et al. Sacituzumab govitecan in metastatic triple-negative breast cancer. N Engl J Med. 2021;384(16):1529–41. 10.1056/NEJMoa2028485. 33882206 10.1056/NEJMoa2028485

[13] Bardia A, Mayer IA, Vahdat LT, Tolaney SM, Isakoff SJ, Diamond JR, O’Shaughnessy J, Moroose RL, Santin AD, Abramson VG, Shah NC, Rugo HS, Goldenberg DM, Sweidan AM, Iannone R, Washkowitz S, Sharkey RM, Wegener WA, Kalinsky K. Sacituzumab Govitecan-hziy in refractory metastatic Triple-Negative breast cancer. N Engl J Med. 2019;380(8):741–51. 10.1056/NEJMoa1814213. Epub 2019/02/21. 30786188 10.1056/NEJMoa1814213

[14] Seligson JM, Patron AM, Berger MJ, Harvey RD, Seligson ND. Sacituzumab Govitecan-hziy: an antibody-drug conjugate for the treatment of refractory, metastatic, triple-negative breast cancer. Ann Pharmacother. 2021;55(7):921–31. 10.1177/1060028020966548. 33070624 10.1177/1060028020966548

[15] Carey LA. Directed therapy of subtypes of triple-negative breast cancer. Oncologist. 2011;16(Suppl 1):71–8. 10.1634/theoncologist.2011-S1-71. 21278443 10.1634/theoncologist.2011-S1-71

[16] Espinosa Fernandez JR, Eckhardt BL, Lee J, Lim B, Pearson T, Seitz RS, et al. Identification of triple-negative breast cancer cell lines classified under the same molecular subtype using different molecular characterization techniques: implications for translational research. PLoS One. 2020;15(4):e0231953. 10.1371/journal.pone.0231953. 32353087 10.1371/journal.pone.0231953 PMC7192374

[17] Mayer IA, Abramson VG, Lehmann BD, Pietenpol JA. New strategies for triple-negative breast cancer–deciphering the heterogeneity. Clin Cancer Res. 2014;20(4):782–90. 10.1158/1078-0432.CCR-13-0583. 24536073 10.1158/1078-0432.CCR-13-0583 PMC3962777

[18] Metzger-Filho O, Tutt A, de Azambuja E, Saini KS, Viale G, Loi S, et al. Dissecting the heterogeneity of triple-negative breast cancer. J Clin Oncol. 2012;30(15):1879–87. 22454417 10.1200/JCO.2011.38.2010

[19] Roncato F, Rruga F, Porcu E, Casarin E, Ronca R, Maccarinelli F, et al. Improvement and extension of anti-EGFR targeting in breast cancer therapy by integration with the avidin-nucleic-acid-nano-assemblies. Nat Commun. 2018;9(1):4070. 10.1038/s41467-018-06602-6. 30287819 10.1038/s41467-018-06602-6 PMC6172284

[20] Criscitiello C. Tumor-associated antigens in breast cancer. Breast Care. 2012;7(4):262–6. 10.1159/000342164. 23904827 10.1159/000342164 PMC3515784

[21] Armstrong JS. Mitochondria: a target for cancer therapy. Br J Pharmacol. 2006;147(3):239–48. 10.1038/sj.bjp.0706556. 16331284 10.1038/sj.bjp.0706556 PMC1751297

[22] Honda HM, Korge P, Weiss JN. Mitochondria and ischemia/reperfusion injury. Ann N Y Acad Sci. 2005;1047:248–58. 10.1196/annals.1341.022. 16093501 10.1196/annals.1341.022

[23] Chen K, Ernst P, Sarkar A, Kim S, Si Y, Varadkar T, et al. mLumiOpto is a mitochondrial-targeted gene therapy for treating cancer. Cancer Res. 2024;84(23):4049–65. 10.1158/0008-5472.CAN-24-0984. 39288077 10.1158/0008-5472.CAN-24-0984 PMC11609628

[24] Chen R, Jiang X, Sun D, Han G, Wang F, Ye M, et al. Glycoproteomics analysis of human liver tissue by combination of multiple enzyme digestion and hydrazide chemistry. J Proteome Res. 2009;8(2):651–61. 10.1021/pr8008012. 19159218 10.1021/pr8008012

[25] Pardoll DM. The blockade of immune checkpoints in cancer immunotherapy. Nat Rev Cancer. 2012;12(4):252–64. 10.1038/nrc3239. 22437870 10.1038/nrc3239 PMC4856023

[26] Vigdorovich V, Ramagopal UA, Lazar-Molnar E, Sylvestre E, Lee JS, Hofmeyer KA, Zang X, Nathenson SG, Almo SC. Structure and T cell Inhibition properties of B7 family member, B7-H3. Structure. 2013;21(5):707–17. PubMed PMID: 23583036; PMCID: PMC3998375. 23583036 10.1016/j.str.2013.03.003 PMC3998375

[27] Chen C, Shen Y, Qu QX, Chen XQ, Zhang XG, Huang JA. Induced expression of B7-H3 on the lung cancer cells and macrophages suppresses T-cell mediating anti-tumor immune response. Exp Cell Res. 2013;319(1):96–102. 10.1016/j.yexcr.2012.09.006. 22999863 10.1016/j.yexcr.2012.09.006

[28] Sun J, Guo YD, Li XN, Zhang YQ, Gu L, Wu PP, Bai GH, Xiao Y. B7-H3 expression in breast cancer and upregulation of VEGF through gene silence. Onco Targets Ther. 2014;7:1979–86. PubMed PMID: 25378933; PMCID: PMC4218908. 25378933 10.2147/OTT.S63424 PMC4218908

[29] Bachawal SV, Jensen KC, Wilson KE, Tian L, Lutz AM, Willmann JK. Breast cancer detection by B7-H3-targeted ultrasound molecular imaging. Cancer Res. 2015;75(12):2501–9. 10.1158/0008-5472.CAN-14-3361. 25899053 10.1158/0008-5472.CAN-14-3361 PMC4470725

[30] Liu C, Liu J, Wang J, Liu Y, Zhang F, Lin W, et al. B7-H3 expression in ductal and lobular breast cancer and its association with IL-10. Mol Med Rep. 2013;7(1):134–8. 10.3892/mmr.2012.1158. 23128494 10.3892/mmr.2012.1158

[31] Zhou ZZ, Si Y, Zhang J, Chen K, George A, Kim S, Zhou L, Liu XM, Dual-Payload A. Antibody-Drug Conjugate Targeting CD276/B7-H3 Elicits Cytotoxicity and Immune Activation in Triple-Negative Breast Cancer. Cancer Res. 2024;84(22):3848-63. Epub 2024/08/26. 10.1158/0008-5472.CAN-23-4099. PubMed PMID: 39186778; PMCID: PMC11565169 the study, as well as a patent for PCT/US2024/037838 pending and a patent for PCT/US2024/037844 pending. X.M. Liu reports grants from NIH and Department of Defense and nonfinancial support from the Ohio State University during the conduct of the study, as well as a patent for PCT/US2024/037838 pending and a patent for PCT/US2024/037844 pending. No disclosures were reported by the other authors. 10.1158/0008-5472.CAN-23-4099 PMC11565169 39186778

[32] N PE, L Z. Precisely control mitochondrial membrane potential with light to manipulate cell fate decisions. Biophys J. 2019;117(4):197–203. 10.1016/j.bpj.2019.06.038 PMC6712847 31400914

[33] Nowsheen S, Bonner JA, LoBuglio AF, Trummell H, Whitley AC, Dobelbower MC, et al. Cetuximab augments cytotoxicity with Poly (ADPRibose) polymerase Inhibition in head and neck cancer. PLoS One. 2011;6(8):e24148. 21912620 10.1371/journal.pone.0024148 PMC3166164

[34] Nowsheen S, Cooper T, Stanley JA, Yang ES. Synthetic lethal interactions between EGFR and PARP inhibition in human triple negative breast cancer cells. PLoS One. 2012;7(10):e46614. 23071597 10.1371/journal.pone.0046614 PMC3469581

[35] Stringer-Reasor EM, May JE, Olariu E, Caterinicchia V, Li Y, Chen D, et al. An open-label, pilot study of veliparib and lapatinib in patients with metastatic, triple-negative breast cancer. Breast Cancer Res. 2021;23(1):30. 10.1186/s13058-021-01408-9. 33663560 10.1186/s13058-021-01408-9 PMC7934554

[36] Al-Ejeh F, Shi W, Miranda M, Simpson PT, Vargas AC, Song S, et al. Treatment of triple-negative breast cancer using anti-EGFR-directed radioimmunotherapy combined with radiosensitizing chemotherapy and PARP inhibitor. J Nucl Med. 2013;54(6):913–21. 10.2967/jnumed.112.111534. 23564760 10.2967/jnumed.112.111534

[37] El Guerrab A, Bamdad M, Kwiatkowski F, Bignon YJ, Penault-Llorca F, Aubel C. Anti-EGFR monoclonal antibodies and EGFR tyrosine kinase inhibitors as combination therapy for triple-negative breast cancer. Oncotarget. 2016;7(45):73618–37. 10.18632/oncotarget.12037. 27655662 10.18632/oncotarget.12037 PMC5342003

[38] Chen K, Si Y, Guan JS, Zhou Z, Kim S, Kim T, et al. Targeted extracellular vesicles delivered verrucarin A to treat glioblastoma. Biomedicines. 2022;10(1):130. 10.3390/biomedicines10010130. 35052809 10.3390/biomedicines10010130 PMC8773723

[39] Si Y, Chen K, Ngo HG, Guan JS, Totoro A, Zhou Z, et al. Targeted EV to deliver chemotherapy to treat triple-negative breast cancers. Pharmaceutics. 2022;14(1):146. 10.3390/pharmaceutics14010146. 35057042 10.3390/pharmaceutics14010146 PMC8781632

[40] Guan JS, Chen K, Si Y, Kim T, Zhou Z, Kim S, Zhou L, Liu XM. Process improvement of adeno-associated virus (AAV) production. Front Chem Eng. 2022;4. Epub 2022/06/11. doi: 10.3389/fceng.2022.830421. PubMed PMID: 35685827; PMCID: PMC9176270. 10.3389/fceng.2022.830421 PMC9176270 35685827

[41] Si Y, Kim S, Zhang E, Tang Y, Jaskula-Sztul R, Markert JM, Chen H, Zhou L, Liu XM. Targeted exosomes for drug delivery: Biomanufacturing, surface Tagging, and validation. Biotechnol J. 2020;15(1):e1900163. 10.1002/biot.201900163. Epub 20191020. 31595685 10.1002/biot.201900163

[42] Si Y, Guan J, Xu Y, Chen K, Kim S, Zhou L, et al. Dual-targeted extracellular vesicles to facilitate combined therapies for neuroendocrine cancer treatment. Pharmaceutics. 2020. 10.3390/pharmaceutics12111079. 33187322 10.3390/pharmaceutics12111079 PMC7696983

[43] Ou J, Si Y, Gah KY, Song J, Patrick E, Flanigan D, Wang L, Zhou L, Liu R, Liu XM. Process development of antibody-drug conjugation production for cancer treatment. PLoS ONE. 2018;e0206246. 10.1371/journal.pone.0206246(13(10). 10.1371/journal.pone.0206246 PMC6198984 30352095

[44] Si Y, Kim S, Ou J, Lu Y, Ernst P, Chen K, et al. Anti-SSTR2 antibody-drug conjugate for neuroendocrine tumor therapy. Cancer Gene Ther. 2020. 10.1038/s41417-020-0196-5. 32684623 10.1038/s41417-020-0196-5 PMC7854894

[45] Si Y, Zhang Y, Guan JS, Ngo H, Totoro A, Singh A, Chen K, Xu Y, Yang E, Zhou L, Liu R, Liu X. Anti-CD47 Monoclonal Antibody-drug Conjugate: A Targeted Therapy to Treat Triple-negative Breast Cancers. Vaccines. 2021. 10.3390/vaccines9080882. 10.3390/vaccines9080882 PMC8402537 34452008

[46] Xu N, Ma C, Ou J, Sun WW, Zhou L, Hu H, et al. Comparative proteomic analysis of three Chinese hamster ovary (CHO) host cells. Biochem Eng J. 2017;124:122–9. 10.1016/j.bej.2017.05.007. 28736500 10.1016/j.bej.2017.05.007 PMC5518618

[47] Xu N, Ou J, Si Y, Goh K, Flanigan D, Yang Y, Yang ST, Zhou L, Liu X. Proteomics insight into the production of monoclonal antibody. Biochem Eng J. 2018. 10.1016/j.bej.2019.02.022.29977134

[48] Chen K, Si Y, Ou J, Guan JS, Kim S, Ernst P, Zhang Y, Zhou L, Han X, Liu XM. Antibody-Drug conjugate to treat meningiomas. Pharmaceuticals (Basel). 2021;14(5). 10.3390/ph14050427. Epub 2021/06/03. 10.3390/ph14050427 PMC8147502 34063284

[49] Xu N, Ou J, Gilani AK, Zhou L, Liu XM. High-level expression of recombinant IgG1 by CHO K1 platform. Front Chem Sci Eng. 2017;9(3):376–80.

[50] Si Y, Kim S, Zhang E, Tang Y, Jaskula-Sztul R, Markert JM, Chen H, Zhou L, Liu XM. Targeted exosomes for drug delivery: Biomanufacturing, surface Tagging, and validation. Biotechnol J. 2019:e1900163. Epub 2019/10/09. 10.1002/biot.201900163. PubMed PMID: 31595685. 10.1002/biot.201900163 31595685

[51] Chen K, Kim S, Yang S, Varadkar T, Zhou ZZ, Zhang J, et al. Advanced biomanufacturing and evaluation of adeno-associated virus. J Biol Eng. 2024;18(1):15. 10.1186/s13036-024-00409-4. 38360753 10.1186/s13036-024-00409-4 PMC10868095

[52] Zhang J, Zhou ZZ, Chen K, Kim S, Cho IS, Varadkar T, Baker H, Cho JH, Zhou L, Liu XM. A CD276-Targeted Antibody-Drug conjugate to treat Non-Small lung cancer (NSCLC). Cells. 2023;12(19). 10.3390/cells12192393. PubMed PMID: 37830607; PMCID: PMC10572050. Epub 2023/10/13. 10.3390/cells12192393 PMC10572050 37830607

[53] Ernst P, Kim S, Yang Z, Liu XM, Zhou L. Characterization of the far-red fluorescent probe MitoView 633 for dynamic mitochondrial membrane potential measurement. Front Physiol. 2023;14:1257739. 10.3389/fphys.2023.1257739. 37936577 10.3389/fphys.2023.1257739 PMC10627182

[54] Yang S, Liu X. Cell culture process for biologics manufacturing: recent development and trends. J Biopharm Bioprocess. 2013;1(2):133–6.

[55] Wu L, Yi B, Wei S, Rao D, He Y, Naik G, et al. Loss of FOXP3 and TSC1 accelerates prostate cancer progression through synergistic transcriptional and posttranslational regulation of c-MYC. Cancer Res. 2019;79(7):1413–25. 10.1158/0008-5472.CAN-18-2049. 30733194 10.1158/0008-5472.CAN-18-2049 PMC6445690

[56] Valente JFA, Queiroz JA, Sousa F. P53 as the focus of gene therapy: past, present and future. Curr Drug Targets. 2018;19(15):1801–17. 10.2174/1389450119666180115165447. 29336259 10.2174/1389450119666180115165447

[57] Roth JA, Swisher SG, Meyn RE. p53 tumor suppressor gene therapy for cancer. Oncol (Williston Park). 1999;13(10 Suppl 5):148–54. Epub 1999/11/07. PubMed PMID: 10550840. 10550840

[58] Picarda E, Ohaegbulam KC, Zang X, Molecular Pathways. Targeting B7-H3 (CD276) for human cancer immunotherapy. Clin Cancer Res. 2016;22(14):3425–31. 10.1158/1078-0432.CCR-15-2428. Epub 2016/05/22. 27208063 10.1158/1078-0432.CCR-15-2428 PMC4947428

[59] Maeda N, Yoshimura K, Yamamoto S, Kuramasu A, Inoue M, Suzuki N, Watanabe Y, Maeda Y, Kamei R, Tsunedomi R, Shindo Y, Inui M, Tamada K, Yoshino S, Hazama S, Oka M. Expression of B7-H3, a potential factor of tumor immune evasion in combination with the number of regulatory T cells, affects against recurrence-free survival in breast cancer patients. Ann Surg Oncol. 2014;21(Suppl 4):S546–54. 10.1245/s10434-014-3564-2. Epub 2014/02/25. 24562936 10.1245/s10434-014-3564-2 PMC4236607

[60] Singh KK. Mitochondria damage checkpoint in apoptosis and genome stability. FEMS Yeast Res. 2004;5(2):127–32. 10.1016/j.femsyr.2004.04. Epub 2004/10/19. 15489195 10.1016/j.femsyr.2004.04.008

[61] Scheibye-Knudsen M, Fang EF, Croteau DL, Wilson DM 3rd, Bohr VA. Protecting the mitochondrial powerhouse. Trends Cell Biol. 2015;25(3):158–70. PubMed PMID: 25499735; PMCID: PMC5576887. 25499735 10.1016/j.tcb.2014.11.002 PMC5576887

[62] Scheid AD, Beadnell TC, Welch DR. Roles of mitochondria in the hallmarks of metastasis. Br J Cancer. 2021;124(1):124–35. 10.1038/s41416-020-01125-8. 33144695 10.1038/s41416-020-01125-8 PMC7782743

[63] Yu D, Liu C, Guo L. Mitochondrial metabolism and cancer metastasis. Ann Transl Med. 2020;8(14):904. 10.21037/atm.2020.03.42. 32793748 10.21037/atm.2020.03.42 PMC7396750

[64] Krieg S, Fernandes SI, Kolliopoulos C, Liu M, Fendt SM. Metabolic signaling in cancer metastasis. Cancer Discov. 2024;14(6):934–52. 10.1158/2159-8290.CD-24-0174. 38592405 10.1158/2159-8290.CD-24-0174 PMC7616057

[65] Iranmanesh Y, Jiang B, Favour OC, Dou Z, Wu J, Li J, Sun C. Mitochondria’s role in the maintenance of cancer stem cells in glioblastoma. Front Oncol. 2021;11:582694. PubMed PMID: 33692947; PMCID: PMC7937970. 33692947 10.3389/fonc.2021.582694 PMC7937970

[66] Olivier C, Oliver L, Lalier L, Vallette FM. Drug resistance in glioblastoma: the two faces of oxidative stress. Front Mol Biosci. 2020;7:620677. 10.3389/fmolb.2020.620677. Epub 2021/02/16. 33585565 10.3389/fmolb.2020.620677 PMC7873048

[67] Li W, Xu X. Advances in mitophagy and mitochondrial apoptosis pathway-related drugs in glioblastoma treatment. Front Pharmacol. 2023;14:1211719. 10.3389/fphar.2023.1211719. 37456742 10.3389/fphar.2023.1211719 PMC10347406

[68] Tsai YT, Lo WL, Chen PY, Ko CY, Chuang JY, Kao TJ, et al. Reprogramming of arachidonate metabolism confers Temozolomide resistance to glioblastoma through enhancing mitochondrial activity in fatty acid oxidation. J Biomed Sci. 2022;29(1):21. 10.1186/s12929-022-00804-3. 35337344 10.1186/s12929-022-00804-3 PMC8952270

[69] Chien CH, Hsueh WT, Chuang JY, Chang KY. Dissecting the mechanism of Temozolomide resistance and its association with the regulatory roles of intracellular reactive oxygen species in glioblastoma. J Biomed Sci. 2021;28(1):18. 10.1186/s12929-021-00717-7. 33685470 10.1186/s12929-021-00717-7 PMC7938520

[70] Wang D, Tai PWL, Gao G. Adeno-associated virus vector as a platform for gene therapy delivery. Nat Rev Drug Discov. 2019;18(5):358–78. 10.1038/s41573-019-0012-9. 30710128 10.1038/s41573-019-0012-9 PMC6927556

[71] Byrne BJ, Flanigan KM, Matesanz SE, Finkel RS, Waldrop MA, D’Ambrosio ES, Johnson NE, Smith BK, Bonnemann C, Carrig S, Rossano JW, Greenberg B, Lalaguna L, Lara-Pezzi E, Subramony S, Corti M, Mercado-Rodriguez C, Leon-Astudillo C, Ahrens-Nicklas R, Bharucha-Goebel D, Gao G, Gessler DJ, Hwu WL, Chien YH, Lee NC, Boye SL, Boye SE, George LA. Current clinical applications of AAV-mediated gene therapy. Mol Ther. 2025;33(6):2479–516. 10.1016/j.ymthe.2025.04.045. Epub 2025/05/07. 40329530 10.1016/j.ymthe.2025.04.045 PMC12172329

[72] Hacker UT, Bentler M, Kaniowska D, Morgan M, Buning H. Towards clinical implementation of adeno-associated virus (AAV) vectors for cancer gene therapy: current status and future perspectives. Cancers (Basel). 2020. 10.3390/cancers12071889. 32674264 10.3390/cancers12071889 PMC7409174

[73] Kumar SR, Markusic DM, Biswas M, High KA, Herzog RW. Clinical development of gene therapy: results and lessons from recent successes. Mol Ther. 2016;3:16034. 10.1038/mtm.2016.34. 10.1038/mtm.2016.34 PMC4879992 27257611

[74] Mendell JR, Al-Zaidy SA, Rodino-Klapac LR, Goodspeed K, Gray SJ, Kay CN, et al. Current clinical applications of in vivo gene therapy with AAVs. Mol Ther. 2021;29(2):464–88. 10.1016/j.ymthe.2020.12.007. 33309881 10.1016/j.ymthe.2020.12.007 PMC7854298

[75] Whiteley LO. An overview of nonclinical and clinical liver toxicity associated with AAV gene therapy. Toxicol Pathol. 2023;51(7–8):400–4. 10.1177/01926233231201408. 37772805 10.1177/01926233231201408

[76] Dhungel BP, Winburn I, Pereira CDF, Huang K, Chhabra A, Rasko JEJ. Understanding AAV vector immunogenicity: from particle to patient. Theranostics. 2024;14(3):1260–88. 10.7150/thno.89380. 38323309 10.7150/thno.89380 PMC10845199

[77] Meliani A, Boisgerault F, Fitzpatrick Z, Marmier S, Leborgne C, Collaud F, et al. Enhanced liver gene transfer and evasion of preexisting humoral immunity with exosome-enveloped AAV vectors. Blood Adv. 2017;1(23):2019–31. 10.1182/bloodadvances.2017010181. 29296848 10.1182/bloodadvances.2017010181 PMC5728288

[78] Erles K, Sebokova P, Schlehofer JR. Update on the prevalence of serum antibodies (IgG and IgM) to adeno-associated virus (AAV). J Med Virol. 1999;59(3):406–11. 10.1002/(sici)1096-9071(199911)59:3%3C406::aid-jmv22%3E3. Epub 1999/09/29. 10502275 10.1002/(sici)1096-9071(199911)59:3<406::aid-jmv22>3.0.co;2-n

[79] Li C, Narkbunnam N, Samulski RJ, Asokan A, Hu G, Jacobson LJ, Manco-Johnson MJ, Monahan PE. Joint outcome study I. Neutralizing antibodies against adeno-associated virus examined prospectively in pediatric patients with hemophilia. Gene Ther. 2012;19(3):288–94. 10.1038/gt.2011.90. Epub 2011/06/24. 21697954 10.1038/gt.2011.90

[80] Boutin S, Monteilhet V, Veron P, Leborgne C, Benveniste O, Montus MF, et al. Prevalence of serum IgG and neutralizing factors against adeno-associated virus (AAV) types 1, 2, 5, 6, 8, and 9 in the healthy population: implications for gene therapy using AAV vectors. Hum Gene Ther. 2010;21(6):704–12. 10.1089/hum.2009.182. 20095819 10.1089/hum.2009.182

[81] Masuda H, Zhang D, Bartholomeusz C, Doihara H, Hortobagyi GN, Ueno NT. Role of epidermal growth factor receptor in breast cancer. Breast Cancer Res Treat. 2012;136(2):331–45. 10.1007/s10549-012-2289-9. 23073759 10.1007/s10549-012-2289-9 PMC3832208

[82] Nakai K, Hung MC, Yamaguchi H. A perspective on anti-EGFR therapies targeting triple-negative breast cancer. Am J Cancer Res. 2016;6(8):1609–23. Epub 2016/09/21. PubMed PMID: 27648353; PMCID: PMC5004067. 27648353 PMC5004067

[83] Changavi AA, Shashikala A, Ramji AS. Epidermal growth factor receptor expression in triple negative and nontriple negative breast carcinomas. J Lab Physicians. 2015;7(2):79–83. 10.4103/0974-2727.163129. Epub 2015/09/30. 26417156 10.4103/0974-2727.163129 PMC4559633

[84] Zakaria Z, Zulkifle MF, Wan Hasan WAN, Azhari AK, Abdul Raub SH, Eswaran J, Soundararajan M, Syed Husain SNA. Epidermal growth factor receptor (EGFR) gene alteration and protein overexpression in Malaysian triple-negative breast cancer (TNBC) cohort. Onco Targets Ther. 2019;12:7749–56. PubMed PMID: 31571924; PMCID: PMC6759283. 31571924 10.2147/OTT.S214611 PMC6759283

[85] Si Y, Xu Y, Guan J, Chen K, Kim S, Yang ES, et al. Anti-EGFR antibody-drug conjugate for triple-negative breast cancer therapy. Eng Life Sci. 2021;21(1–2):37–44. 10.1002/elsc.202000027. 33531889 10.1002/elsc.202000027 PMC7837297

[86] Wahby S, Fashoyin-Aje L, Osgood CL, Cheng J, Fiero MH, Zhang L, et al. FDA approval summary: accelerated approval of sacituzumab Govitecan-hziy for third-line treatment of metastatic triple-negative breast cancer. Clin Cancer Res. 2021;27(7):1850–4. 10.1158/1078-0432.CCR-20-3119. 33168656 10.1158/1078-0432.CCR-20-3119

[87] McGuinness JE, Kalinsky K. Antibody-drug conjugates in metastatic triple negative breast cancer: a spotlight on sacituzumab govitecan, ladiratuzumab vedotin, and trastuzumab deruxtecan. Expert Opin Biol Ther. 2020(7). 10.1080/14712598.2021.1840547. 10.1080/14712598.2021.1840547 33089726

[88] Isorna I, Gonzalez-Moles MA, Munoz M, Esteban F. Substance P and neurokinin-1 receptor system in thyroid cancer: potential targets for new molecular therapies. J Clin Med. 2023. 10.3390/jcm12196409. 37835053 10.3390/jcm12196409 PMC10573850

[89] Metman MJH, Jonker PKC, Sondorp LHJ, van Hemel BM, Sywak MS, Gill AJ, Jansen L, van Diest PJ, van Ginhoven TM, Lowik C, Nguyen AH, Robinson DJ, van Dam GM, Links TP, Coppes RP, Fehrmann RSN, Kruijff S. MET-receptor targeted fluorescent imaging and spectroscopy to detect multifocal papillary thyroid cancer. Eur J Nucl Med Mol Imaging. 2024;51(8):2384–94. 10.1007/s00259-023-06525-5. Epub 2023/11/29. 38017325 10.1007/s00259-023-06525-5 PMC11178647

[90] Ruco L, Scarpino S. The pathogenetic role of the HGF/c-Met system in papillary carcinoma of the thyroid. Biomedicines. 2014;2(4):263–74. 10.3390/biomedicines2040263. 28548071 10.3390/biomedicines2040263 PMC5344270

[91] Garcia C, Buffet C, El Khattabi L, Rizk-Rabin M, Perlemoine K, Ragazzon B, et al. MET overexpression and activation favors invasiveness in a model of anaplastic thyroid cancer. Oncotarget. 2019;10(23):2320–34. 10.18632/oncotarget.26798. 31040922 10.18632/oncotarget.26798 PMC6481343

