# Supplementary material for: Combine mitochondrial-targeted gene therapy and chemotherapy to treat triple-negative breast cancer
Source: J Exp Clin Cancer Res. 2025 Dec 29;44:328. doi: 10.1186/s13046-025-03572-8 (PMC12750853; doi:10.1186/s13046-025-03572-8)
Supplement: Supplementary file 1 — Supplementary Material 1. Figure S1. Evaluation of potential off-target of our CD276 mAb.Representative IHC images of human normal organs stained with our humanized CD276 mAb. Scale bar equals 70 µm. Figure S2. Evaluation of CD276 expression and tumor-specificity of our CD276 mAb in mouse.Representative IHC images of mouse normal organs stained with our humanized anti-human/mouse CD276 mAb. Mouse malignant mouse adrenal gland was used as control. n=3. Figure S3. Production of humanized CD276 mAb. (A) Production of mAb in 2-L stirred-tank bioreactor at Temp 37oC, Agt 140 rpm, DO 40%, and pH 7.0. (B) Purification using liquid chromatography with Bio-Scale Mini UNOsphere SUPrA affinity column. Loading buffer A: 0.02 M Na3PO4, 0.02 M Na3C6H5O7, pH 7.5. Elution buffer B: 0.1 M NaCl, 0.02 M Na3C6H5O7, pH 3.0. Figure S4. H&E staining of major organs harvested from MDA-MB-231 xenografted NSG mouse models, including brain, heart, lungs, liver, spleen and kidneys. Figure S5. Toxicity assessment in 4T1 metastasis models. (A) IHC staining of lung tissues carrying TNBC metastasis. (B) Body weight profiles. (C) H&E staining of major organs harvested from 4T1 metastatic models, including brain, heart, lungs, liver, spleen and kidneys. Scale bar equals 20 µm. Figure S6. Anti-TNBC efficacy in MDA-MB-231 metastatic models. (A) IVIS imaging of NSG carrying TNBC metastasis. n= 5. Mice were treated with ccmLumiOpto (medium dose, 10x1010ptc/kg-BW)/Olaparib (50 mg/kg), and saline (control). (B) Body weight changes. (C) H&E staining of lung tissues with TNBC metastasis. Figure S7. H&E staining of major organs harvested from PDX models, including brain, heart, lungs, liver, spleen and kidneys. Scale bar equals 20 µm. Figure S8. Complete blood cell count demonstrated low peripheral toxicity of cmLumiOpto/PARPi in BALB/cJ mice. n=2 [file 13046_2025_3572_MOESM1_ESM.docx]

Online Supplemental Materials for

**Combine mitochondrial-targeted gene therapy and chemotherapy to treat triple-negative breast cancer**

Tanvi Varadkar *et al*

Corresponding author: Xiaoguang "Margaret" Liu, [liu.482@osu.edu](mailto:liu.482@osu.edu)

**Supplementray Materials**

**This file includes:**

Figs S1 to S8

**Supplemental Figures**

**
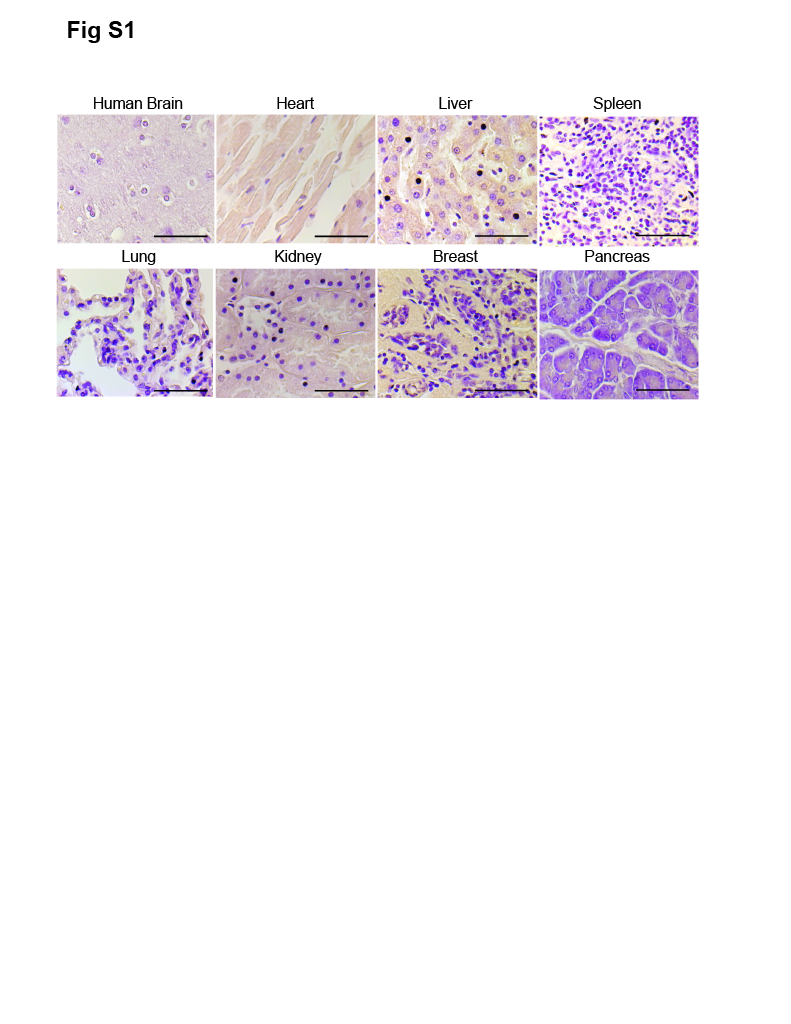
**

**Figure S1. Evaluation of potential off-target of our CD276 mAb.** Representative IHC images of human normal organs stained with our humanized CD276 mAb. Scale bar equals 70 µm.

**
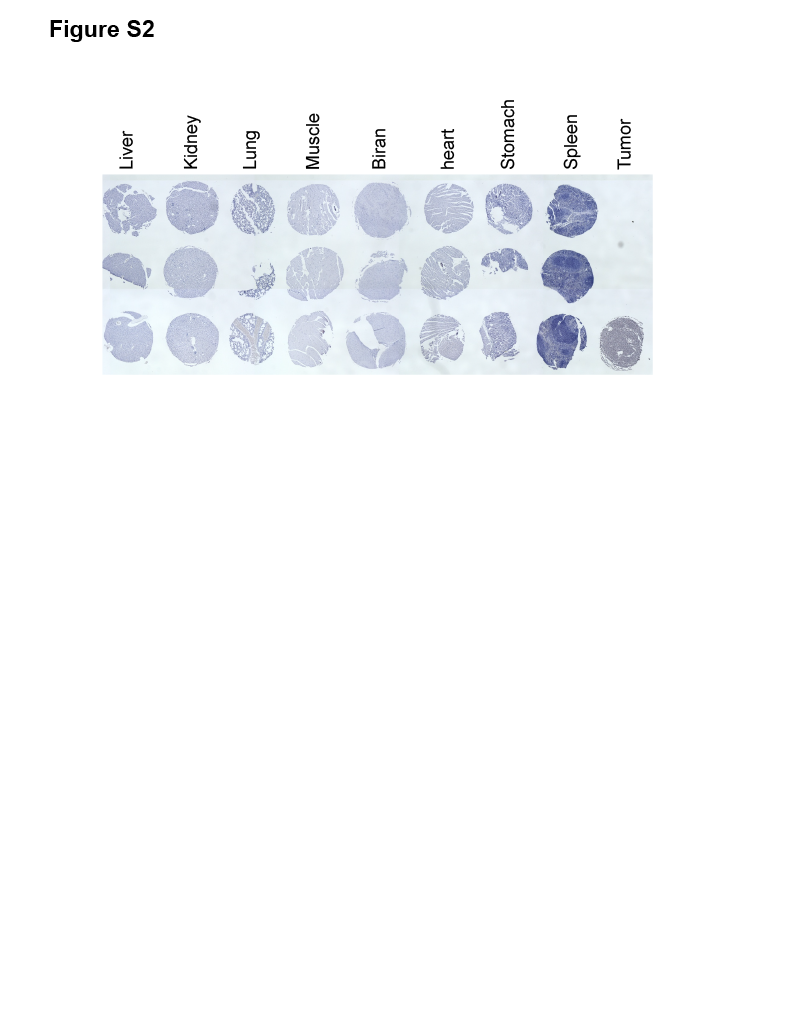
**

**Figure S2. Evaluation of tumor-selectivity of our CD276 mAb in mouse.** Representative IHC images of mouse normal organs stained with our humanized anti-human/mouse CD276 mAb. n=3. Mouse malignancy was used as control.

**
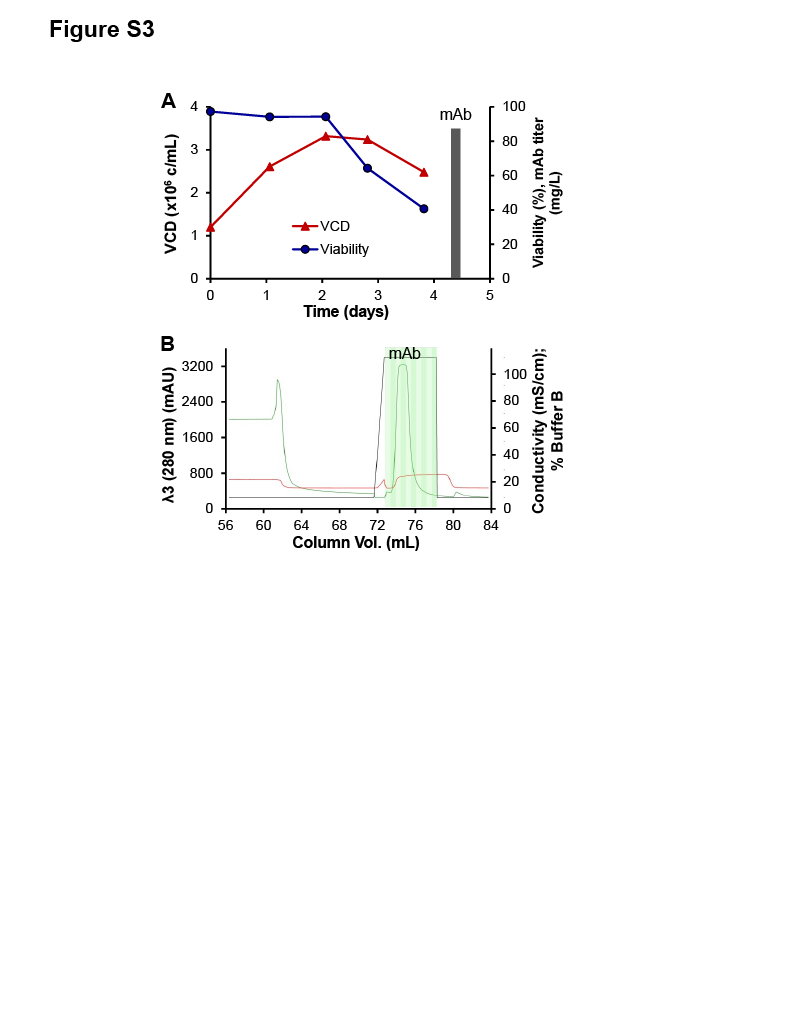
**

**Figure S3. Production of humanized CD276 mAb.** (**A**) Production of mAb in 2-L stirred-tank bioreactor at Temp 37^o^C, Agt 140 rpm, DO 40%, and pH 7.0. (**B**) Purification using liquid chromatography with Bio-Scale Mini UNOsphere SUPrA affinity column. Loading buffer A: 0.02 M Na_3_PO_4_, 0.02 M Na_3_C_6_H_5_O_7_, pH 7.5. Elution buffer B: 0.1 M NaCl, 0.02 M Na_3_C_6_H_5_O_7_, pH 3.0.

**
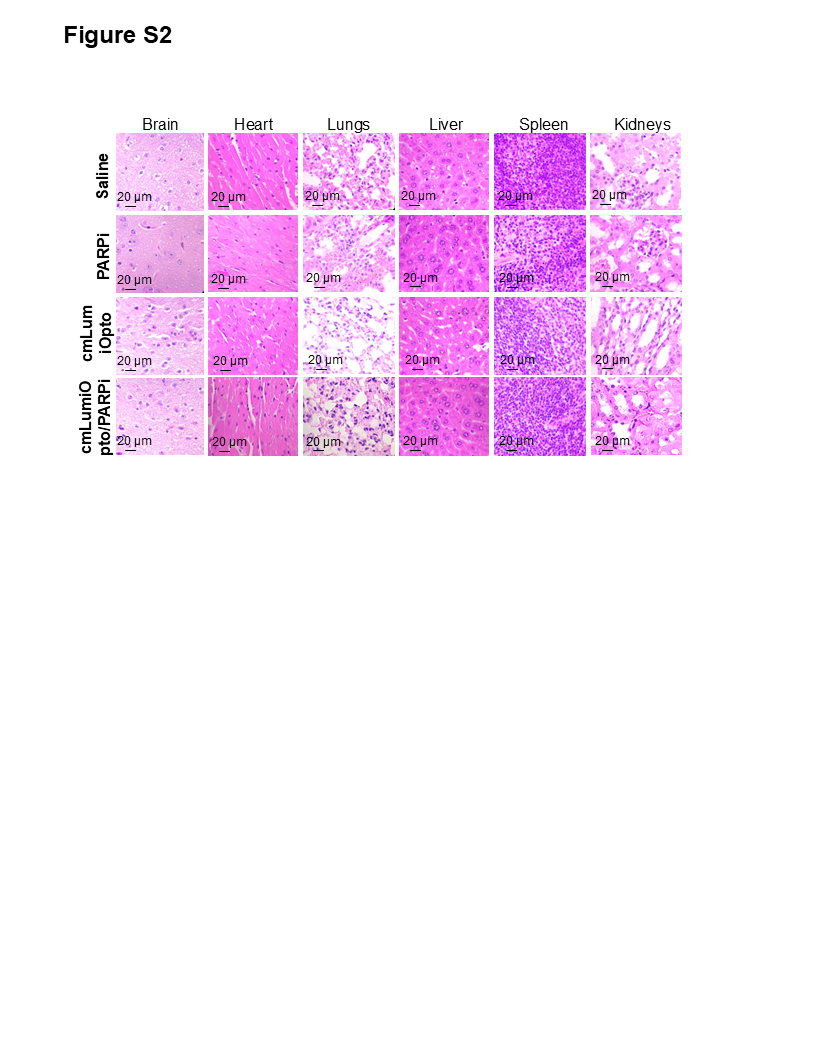
**

**Figure S4. H&E staining of major organs harvested from MDA-MB-231 xenografted NSG mouse models**, including brain, heart, lungs, liver, spleen and kidneys.


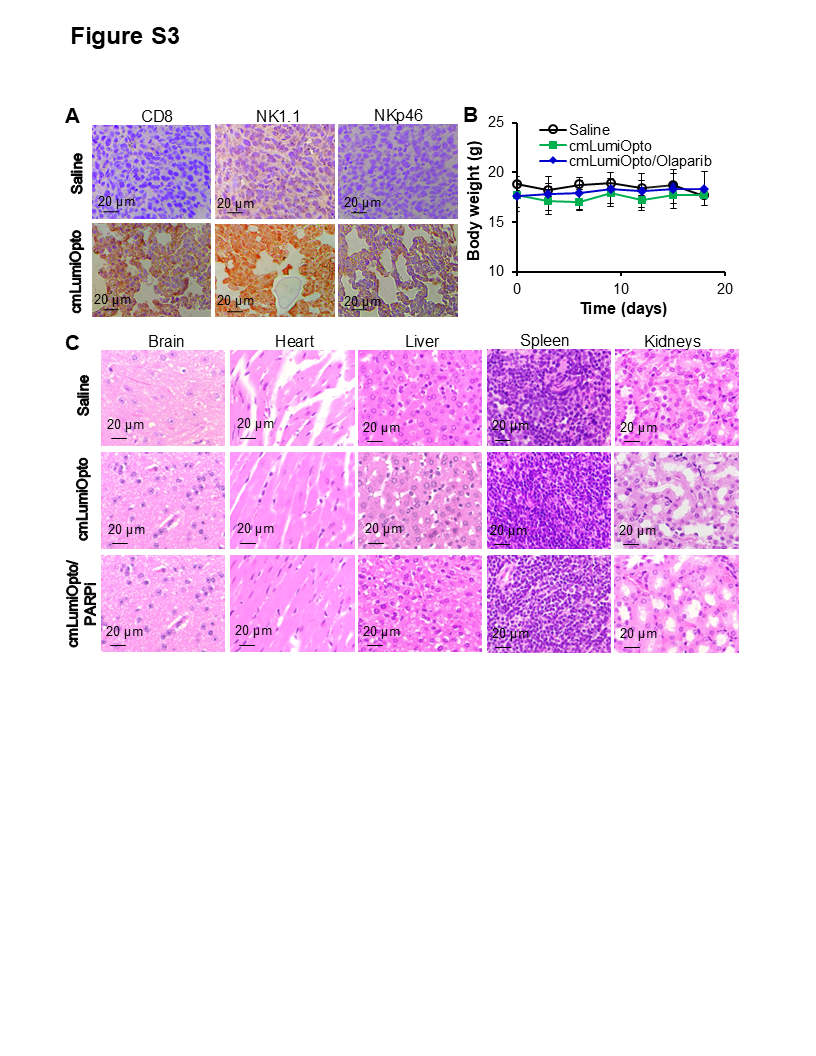


**Figure S5. Toxicity assessment in 4T1 metastasis models.** (**A**) IHC staining of lung tissues carrying TNBC metastasis. (**B**) Body weight profiles. (**C**) H&E staining of major organs harvested from 4T1 metastatic models, including brain, heart, lungs, liver, spleen and kidneys. Scale bar equals 20 µm.


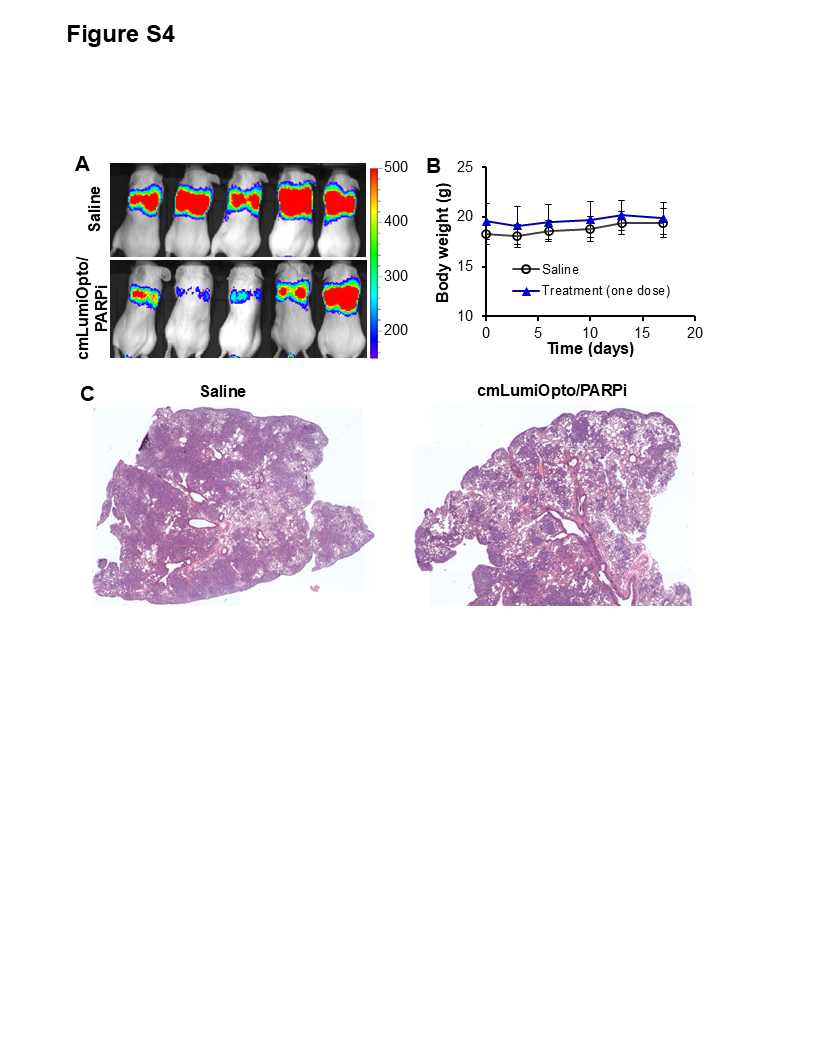


**Figure S6. Anti-TNBC efficacy in MDA-MB-231 metastatic models.** (**A**) IVIS imaging of NSG carrying TNBC metastasis. *n* = 5. Mice were treated with cmLumiOpto (medium dose, 10x10^10^ ptc/kg-BW)/Olaparib (50 mg/kg), and saline (control). (**B**) Body weight changes. (**C**) H&E staining of lung tissues with TNBC metastasis.


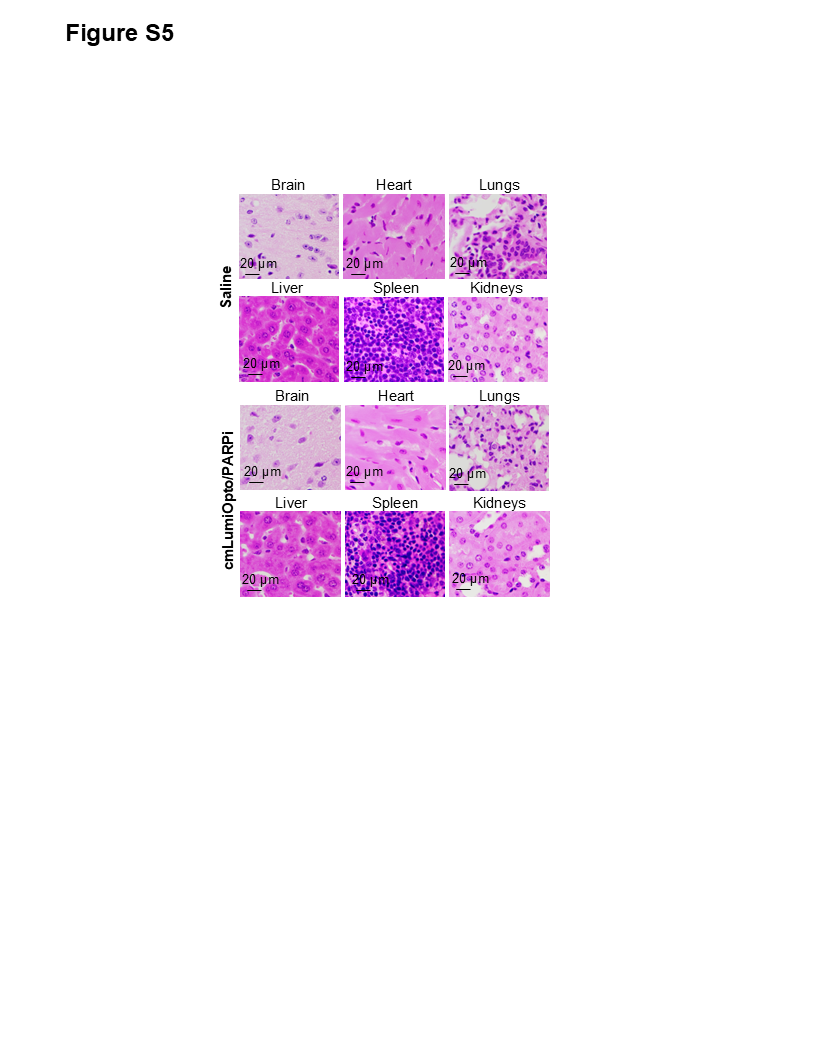


**Figure S7. H&E staining of major organs harvested from PDX models**, including brain, heart, lungs, liver, spleen and kidneys. Scale bar equals 20 µm.


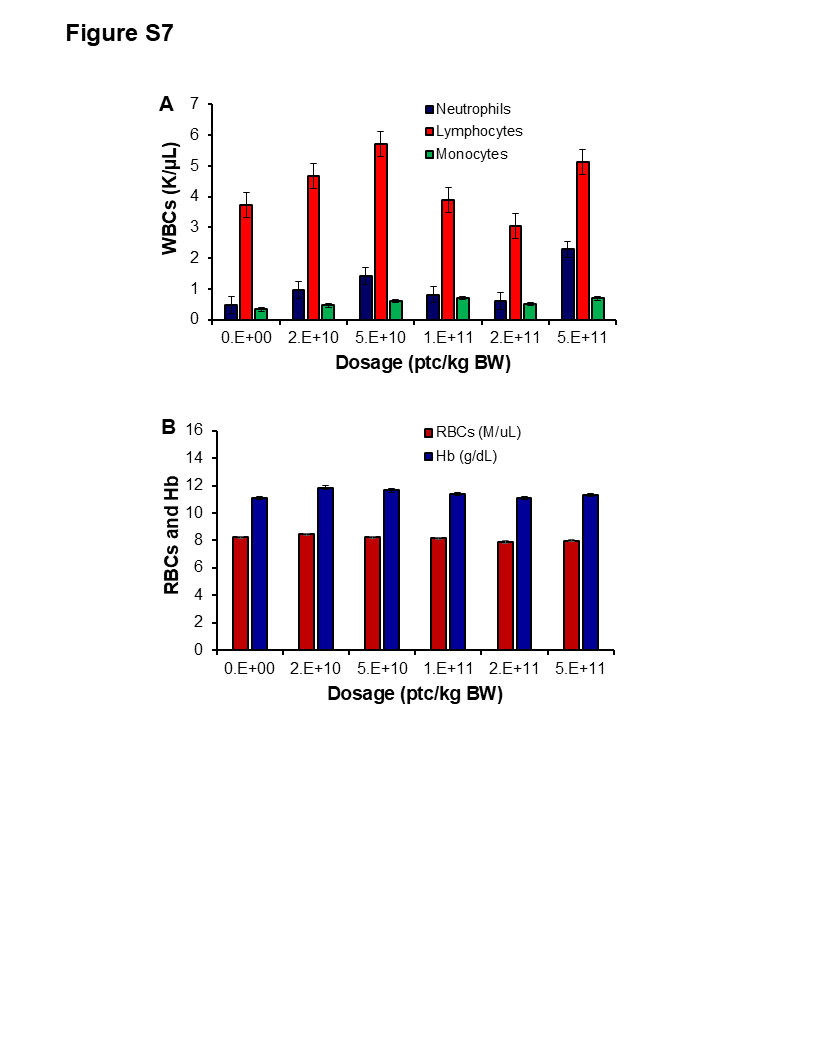


**Figure S8. Complete blood cell count** demonstrated low peripheral toxicity of cmLumiOpto/PARPi in BALB/cJ mice. n=2.
